# A BRCT domain-containing protein induced in early phagocytosis plays a crucial role in the pathogenesis of the mucoralean *Rhizopus microsporus*

**DOI:** 10.1371/journal.ppat.1013653

**Published:** 2026-01-02

**Authors:** Ghizlane Tahiri, Carlos Lax, Ulrike Binder, Jakob Scheler, Eusebio Navarro, Francisco E. Nicolás, Victoriano Garre

**Affiliations:** 1 Departamento de Genética y Microbiología, Facultad de Biología, Universidad de Murcia, Murcia, Spain; 2 Department of Hygiene, Microbiology and Public Health, Division of Hygiene and Medical Microbiology, Medical University Innsbruck, Innsbruck, Austria; Texas Tech University Health Sciences Center School of Medicine - Lubbock Campus: Texas Tech University Health Sciences Center School of Medicine, UNITED STATES OF AMERICA

## Abstract

Mucormycosis, caused by mucoralean fungi, is among the most lethal fungal diseases, and a deeper understanding of its pathogenesis is urgently needed. Transcriptomic profiling of virulent strain (WT) and an RNAi-deficient strain (*r3b2*Δ) of *M. lusitanicus* during phagocytosis uncovered thousands of differentially expressed genes (DEGs), highlighting early metabolic activation as a key survival strategy inside the phagosome. Enriched pathways included amino acid transport, nucleotide metabolism, and translation, reflecting an adaptive fungal response to nutrient deprivation and host immune stress. Integrative analyses of mRNA and sRNA profiles also revealed a critical role of the RNAi pathways in modulating gene expression during infection. Building on these observations, we identified four chromatin- and transcription-related candidate virulence genes, *brca1*, *box*, *hist1*, and *hda10*, which were strongly upregulated during phagocytosis and regulated by RNAi. Functional disruption of their orthologs in the clinically relevant pathogen *R. microsporus* significantly reduced virulence in healthy mice, particularly in the *brca1, hda1,* and *hist1* mutants. The *brca1* knockout mutant also exhibited lower fungal burden in the brain and lungs and reduced survival rates after exposure to peritoneal immune cells. In contrast, their deletion in *M. lusitanicus* had no detectable impact on virulence in healthy animal models, highlighting species-dependent differences in pathogenic potential. These results demonstrate that *M. lusitanicus* is a valuable genetic model. However, combining studies across multiple Mucorales species is essential to uncover both conserved and species-specific mechanisms of host adaptation and virulence. These insights contribute to a broader understanding of fungal adaptation, immune evasion, and the identification of novel targets for antifungal intervention.

## Author summary

Mucormycosis is a severe fungal infection caused by Mucorales species, often affecting immunocompromised individuals. These pathogens can survive inside macrophages, immune cells that normally destroy invading microbes. Understanding how Mucorales escape these defenses is essential for developing new treatments. In this study, we investigated early interactions between macrophages and the model organism *Mucor lusitanicus*. Analysis of gene expression during phagocytosis revealed activation of metabolic and stress-response pathways that promote intracellular survival, with RNA interference contributing to their regulation. Importantly, we identified several chromatin- and transcription-related genes; *brca1*, *hist1*, *hda10*, and *box*, that are induced during host–pathogen interactions. Functional experiments showed that loss of these genes in *Rhizopus microsporus*, one of the main causal agents of mucormycosis, reduced its ability to cause disease in mice. In particular, loss of the *brca1* gene strongly impaired virulence, resulting in reduced fungal burden in the lungs and brain. It also decreases survival following exposure to peritoneal immune cells. In contrast, deletion of these genes in *M. lusitanicus* did not affect virulence. These findings highlight species-specific differences in virulence mechanisms. Overall, our work provides new insights into fungal adaptation to host immunity, identifying potential antifungal targets.

## Introduction

Mucoralean fungi, such as *Mucor* and *Rhizopus* species, are opportunistic pathogens responsible for mucormycosis, a severe and often fatal infection that primarily affects immunocompromised individuals [1]. Infection occurs mainly through inhalation of spores, which deposit in the paranasal sinuses and lungs, although ingestion or direct inoculation through the skin can also occur [1,2]. Under normal conditions, tissue invasion is largely prevented by physical barriers and innate immune defenses. Alveolar macrophages, which closely interact with the respiratory epithelium, play a central role in controlling spore germination and early fungal growth [3]. In immunocompromised patients, these defenses can be impaired, allowing Mucorales to invade multiple tissues, including the lungs, central nervous system, paranasal sinuses, gastrointestinal tract, and skin [4].

Among the innate immune effectors, macrophages play a central role in restricting Mucorales infection. These phagocytes are rapidly recruited to infection sites, where they recognize pathogen-associated molecular patterns (PAMPs) via pattern recognition receptors (PRRs) and internalize fungal spores into phagosomes. These then fuse with lysosomes to form acidic phagolysosomes, exposing the spores to nitric oxide and nutrient deprivation, particularly iron restriction, ultimately inhibiting germination and promoting pathogen clearance [5].

However, some of these fungal pathogens have evolved mechanisms to evade or manipulate this immune response, ensuring their survival inside phagocytes. The ability of *Mucorales* to cause infection relies on a broad arsenal of virulence factors. For example, in *M. lusitanicus,* the high-affinity iron-uptake system (*fet3a*, *fet3b*, *fet3c*, *ftr1*) is essential for growth and pathogenicity, with *fet3c* playing a particularly critical role [6]. Additionally, *M. lusitanicus* secretes the siderophore rhizoferrin, which contributes to virulence by facilitating iron acquisition under host-imposed nutrient limitation [7]. In *Rhizopus delemar,* the high-affinity iron-uptake system is also indispensable for virulence [8]. Interestingly, cell wall antigens, such as the CotH protein family, mediate host adhesion and immune evasion in *R. delemar* [9] and in *M. lusitanicus* [10]. Moreover, *R. delemar* produces the ricin-like toxin mucoricin, a secreted virulence factor that promotes extensive tissue necrosis and accelerates disease progression [11]. Together, these factors illustrate how Mucorales employ complementary strategies, ranging from epithelial invasion and nutrient acquisition to immune evasion, that collectively support infection.

Importantly, these pathogens also display unique adaptations to resist or exploit phagocytosis, thereby promoting intracellular persistence and dissemination*. In vitro* studies indicate that while macrophages can recognize and phagocytose Mucorales spores, certain species can germinate intracellularly, thereby surviving the host defense mechanisms [12]. For instance, *Rhizopus* species can arrest phagosome maturation, preventing macrophage-mediated killing [13]. Additionally, *Rhizopus microsporus* harbors the bacterial endosymbiont *Ralstonia pickettii*, which secretes factors that impair the ability of the host to detect and eliminate the fungal pathogen [14]. Similarly, *M. lusitanicus* spores resist intracellular elimination by blocking phagosome maturation through a calcineurin-dependent mechanism, which promotes their survival and germination within macrophages [15]. This ability to grow inside host cells is partially regulated by the transcription factors Atf1 and Atf2 [6]. Furthermore, in *M. lusitanicus*, spore size influences virulence, as only larger spores can germinate inside macrophages. Interestingly, *M. lusitanicus* induces apoptosis in macrophages but not neutrophils during zebrafish infections [16].

During the interaction between Mucorales and their hosts, extensive genetic regulatory changes occur, including modulation of gene expression by RNA interference (RNAi). In *M. lusitanicus*, two major RNAi pathways have been described: the canonical RNAi pathway and the non-canonical RNAi Pathway (NCRIP) [17]. The canonical pathway depends on Dicer and Argonaute proteins to process double-stranded RNA (dsRNA) into small interfering RNAs (siRNAs), which regulate gene expression and maintain genome stability [18,19].

In contrast, NCRIP functions independently of Dicer and Argonaute. Instead, it relies on the RNase III-like enzyme R3B2, which specifically cleaves single-stranded RNAs (ssRNAs), along with RNA-dependent RNA polymerases (RdRPs) [6]. This pathway plays a crucial role in controlling gene expression under stress conditions, including host-pathogen interactions. Studies have shown that NCRIP is involved in fungal virulence, as its disruption alters stress responses and reduces pathogenicity, suggesting its importance in infection and survival within the host [18].

Although previous studies have explored RNAi pathways in Mucorales pathogenesis, they largely focused on later stages of phagocytosis after spore germination [18]. Here, we aimed to characterize the earliest phases of macrophage interaction by integrating transcriptome and degradome, through mRNA and sRNA analyses. This approach revealed coordinated control by canonical and NCRIP pathways, with *M. lusitanicus* modulating gene degradation in response to phagocytosis, highlighting molecular responses critical for fungal fitness and host adaptation. Furthermore, this study investigates the role of conserved virulence factors in *M. lusitanicus* and *R. microsporus* by analyzing early phagocytosis events using mutant strains and infection models. Through functional analysis, we identified the Histone H1 (*hist1*) ortholog as a key contributor to fungal fitness and the *brca1* ortholog as a determinant of pathogenesis. These findings provide novel insights into the genetic basis of virulence attenuation in *R. microsporus*, shedding light on conserved mechanisms of immune evasion during the initial stages of host-pathogen interaction.

## Results

### *M. lusitanicus* deploys a major transcriptional response in early phagocytosis

**Fig 1:**
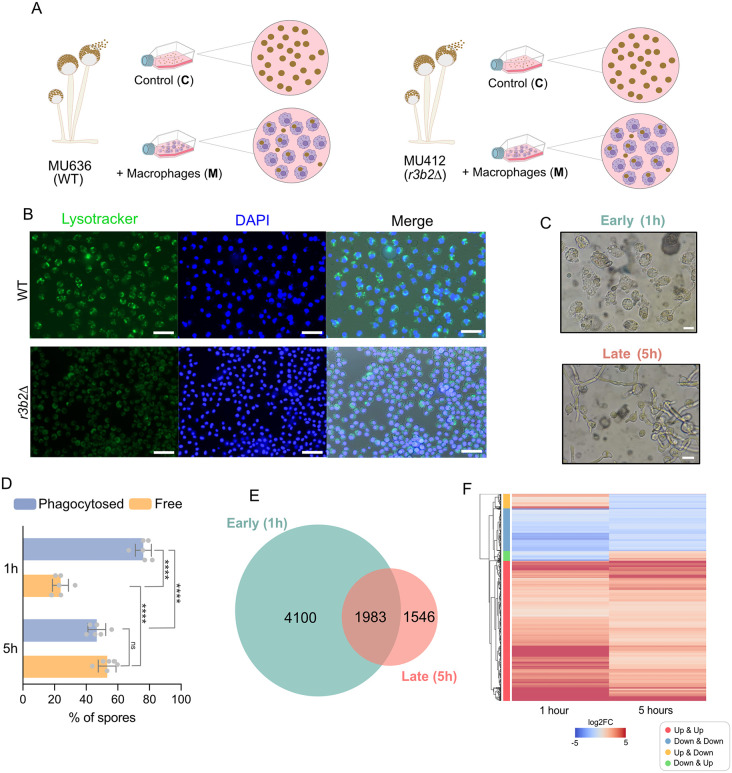
(A) Schematic representation of the RNA-seq analysis experimental design, including macrophage phagocytosis and saprophytic conditions. The assays included wild-type (WT) spores with and without macrophages (WT-M and WT-C), and *r3b2*Δ spores with and without macrophages (*r3b2*Δ-M and *r3b2*Δ-C). (B) Fluorescence microscopy of macrophages stained with LysoTracker Green DND-26 (green) and Hoechst 3342 (blue) during early interaction with *Mucor* spores from WT and *r3b2*Δ strains (scale bar = 15 µm). (C) Light microscopy images showing the interaction between *Mucor* spores and macrophages at early (1 h) and late (5 h) time points (scale bar = 50 µm). (D) Quantification of internalized versus free spores during early and late stages of phagocytosis. Data are represented as mean ± SD. Statistical significance: **** p < 0.0001 (Welch’s *t*-*t*est). (E) Comparative transcriptomic analysis of the WT *Mucor* strain during early and late phagocytosis. (F) Differentially expressed genes (DEGs) shared between early and late phagocytosis, classified by expression patterns: red indicates genes upregulated at both time points; blue, downregulated at both; orange, upregulated early and downregulated late; and green, downregulated early and upregulated late.

To determine the transcriptional profile of *M. lusitanicus* spores during early phagocytosis, a time-course experiment was conducted at different intervals of macrophage internalization (30 min, 60 min, 90 min, 2 h, 3 h, and 5 h). The goal was to identify the most suitable time point at which a more substantial number of *M. lusitanicus* spores are phagocytosed, ensuring sufficient fungal RNA concentration for analysis. Two *M. lusitanicus* strains were used to monitor phagocytosis: a wild-type strain (WT, MU636) and a knockout mutant strain in the *r3b2* gene (MU412). The internalization process was analyzed using an *in vitro* host-pathogen system, where both strains were confronted with the J774A.1 mouse macrophages cell line (ATCCT1B-G7) at a ratio of 1.5:1, respectively during 1 h. As non-interaction controls, fungal spores from both strains were grown under saprophytic conditions in L15 medium for the same time (Fig 1A-B). Additionally, mRNAs and sRNAs were extracted at each time point, as well as from the non-interaction controls to assess RNA availability.

Quantification of spore internalization showed that nearly all *M. lusitanicus* spores were phagocytosed by 1 h post-infection (p < 0.0001, Welch’s t-test), with significant differences between phagocytosed and free spores at 1 h, and between early (1 h) and late (5 h) time points (Fig 1C–D). The apparent increase in free spores at 5 h reflected fungal germination within phagolysosomes and hyphal escape rather than reduced uptake (Fig 1C–D). Combined with the detection of fungal RNA at all time points, these observations indicated that 1 h post-infection provides an optimal window to capture early transcriptional responses for RNA-seq, prior to hyphal emergence. Importantly, this early stage was selected to identify RNAi-related target genes, as previous studies have mainly focused on later growth phases [19].

To investigate the transcriptional dynamics of *M. lusitanicus* during phagocytosis, we compared the differentially expressed genes (DEGs) of the WT strain at each time point relative to its own saprophytic control, after 1 hour (early phagocytosis) and 5 hours (late phagocytosis) of interaction. The 5 h dataset was obtained from our previous work [18], allowing direct comparison between early and late transcriptional responses.

Interestingly, we identified 4100 genes that are exclusively differentially expressed during early phagocytosis, whereas only 1546 genes are uniquely expressed in the late phase (Fig 1E). These findings suggest that *M. lusitanicus* mounts a major transcriptional response during the initial stages of phagocytosis. The transcriptional profile comparison further revealed that 1983 genes are differentially expressed at both 1 hour and 5 hours post-phagocytosis (Fig 1F; S1 File). Among these, a subset of genes exhibits a chronological transcriptional response, with 1419 genes (Fig 1F, red) consistently upregulated and 473 genes (Fig 1F, blue) persistently downregulated at both time points. Additionally, some genes display a temporal activation specific to either the early or late phase of phagocytosis: 114 genes undergo transient upregulation at 1 hour followed by downregulation at 5 hours (Fig 1F, orange), whereas 106 genes follow the opposite pattern, being downregulated at 1 hour and upregulated at 5 hours (Fig 1F, green).

### Transcriptional response of *M. lusitanicus* to early phagocytosis

**Fig 2:**
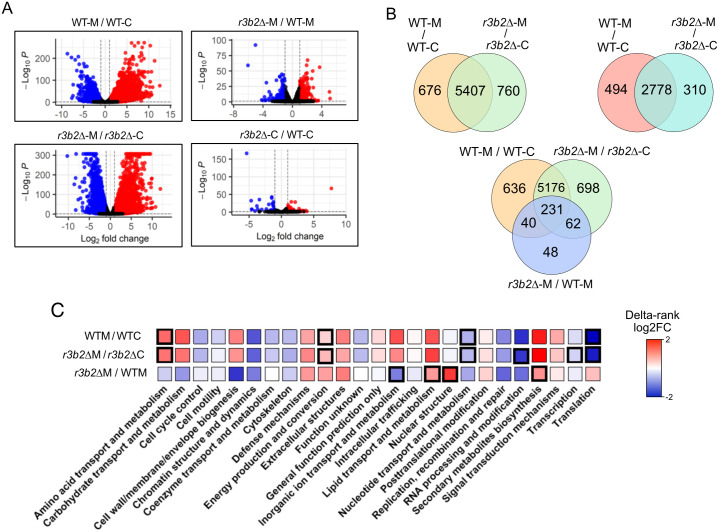
Early transcriptomic changes of the fungal spores during their phagocytosis by macrophages. **(A)** Volcano plots of the WT strain fungal spores with and without macrophages (WT-M/WT-C), the *r3b2∆* versus the WT interacting with macrophages (*r3b2∆*-M/ WT-M), the *r3b2∆* with and without macrophages (*r3b2∆*-M/ *r3b2∆*-C), and the *r3b2∆* versus the WT in saprophytic conditions (*r3b2∆*-C/ WT-C). Red and blue dots indicate the upregulated and downregulated genes, respectively, while black dots show not differentially expressed genes. **(B)** Venn diagrams illustrating the overlap of DEGs among the different comparisons. The upper left panel shows common DEGs between WT and *r3b2∆* strains during macrophage interaction. The upper right panel shows shared upregulated genes between the macrophage-interacting and saprophytic conditions within each strain. The lower panel (three-set Venn diagram) depicts the intersection of DEGs identified in WT-M versus WT-C, *r3b2∆-*M versus *r3b2∆*, and *r3b2∆* versus WT-M, highlighting genes consistently regulated across conditions. **(C)** Enrichment analysis of the DEG of the WT during its interaction with macrophages versus its saprophytic growth, the enrichment analysis of those DEGs during the interaction of the mutant with macrophages versus its growth under non-stressful conditions, and the enrichment of the DEGs when comparing the mutant versus the WT during their phagocytosis.

To dissect the transcriptional regulation during early phagocytosis, we analyzed both the WT and the RNAi-deficient *r3b2*Δ mutant strains of *M. lusitanicus*. Macrophage interaction triggered extensive transcriptional reprogramming, involving thousands of DEGs in both strains (WT-M versus WT-C and *r3b2*Δ-M versus *r3b2*Δ-C) (Fig 2A; S1 File). The majority of these changes were shared across both strains (Fig 2B), indicating that the global phagocytosis response is largely conserved, while a smaller fraction of genes displayed strain-specific regulation. This suggests that RNAi contributes to fine-tuning rather than completely reshaping the early transcriptional response.

In parallel, we examined whether the NCRIP pathway was active during the earliest stage of the fungal life cycle. Even under non-infectious conditions, NCRIP regulated a distinct subset of genes after short-term growth (*r3b2*Δ-C versus WT-C) (Fig 2A; S1 File), demonstrating that this pathway operates already at the spore stage.

Having demonstrated the activity of the NCRIP pathway during the initial stages of fungal development, specifically at the spore stage, which is the common morphology typically inhaled by hosts, we wanted to assess whether the NCRIP pathway is active during the early phagocytosis of *M. lusitanicus* spores. To that end, we compared the transcriptional profiles of the *r3b2*Δ and WT strains during macrophage interaction (*r3b2*Δ-M versus WT-M). This analysis showed that most transcriptional responses overlapped between both strains (Fig 2B**, upper left panel)**. Interestingly, a subset of genes was commonly upregulated under these conditions (Fig 2B**, upper right panel)**. Furthermore, when integrating all comparisons, including WT-M versus WT-C, *r3b2Δ*-M versus *r3b2Δ*-C, and *r3b2Δ*-M versus WT-M, we identified a core set of genes co-regulated by NCRIP and phagocytosis (Fig 2B**, lower panel)**. Notably, several of these genes were specifically associated with the virulent WT strain, pointing to a potential role of NCRIP in controlling genes linked to invasion and virulence.

To gain functional insight of these gene sets, we performed a KOG analysis (Fig 2C). This revealed that the NCRIP governs key metabolic processes, both under saprophytic conditions and during macrophage interaction, reinforcing its role in metabolic adaptation as a potential virulence strategy. Early phagocytosis itself modulated functions related to amino acid and nucleotide metabolism, energy production, and translation, while processes such as ion and lipid transport, nuclear organization, and secondary metabolite biosynthesis were found to be co-regulated by NCRIP and phagocytosis. These functions are likely to be critical for fungal survival within macrophages and thus represent promising candidates for downstream experimental validation of virulence traits.

### Integrative degradome analysis: sRNA production and mRNA dynamics in the early phagocytosis of *M. lusitanicus*

**Fig 3:**
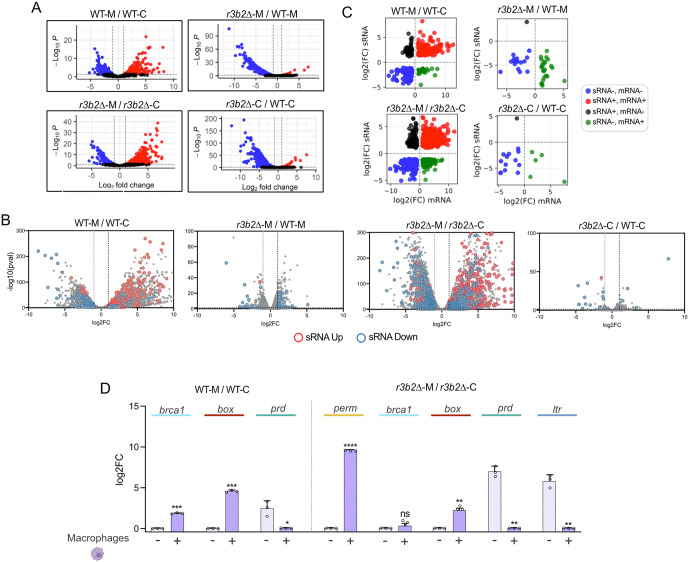
The degradome analysis based on the correspondence between the sRNA production and mRNA expression. **(A)** Volcano plots showing differential sRNA expression in the following comparisons: WT interacting with macrophages versus saprophytic growth (WT-M/WT-C), *r3b2*Δ interacting with macrophages versus WT interacting with macrophages (*r3b2*Δ-M/WT-M), *r3b2*Δ interacting with and without macrophages (*r3b2*Δ-M/r3b2Δ-C), and *r3b2*Δ versus WT under saprophytic conditions (*r3b2*Δ-C/WT-C). **(B)** Analysis of differentially expressed genes (DEGs) from the mRNA dataset based on their corresponding sRNA levels across the following comparisons: WT-M/ WT–C, *r3b2*∆-M/ WT-M, *r3b2*∆-M/ *r3b2*∆–C, and *r3b2*∆–C/ WT–C. The number of upregulated and downregulated sRNAs was determined for each condition. **(C)** Scatter plots illustrating the relationship between the expression of the sRNA and mRNA in the WT-M/ WT-C, *r3b2∆-*M/ WT-M, *r3b2∆-*M/ *r3b2∆-*C, and *r3b2∆-*C/ WT-C. Blue dots indicate genes downregulated at both RNA levels with, black dots are genes upregulated at the sRNA level and downregulation at the mRNA level, the red dots are genes with upregulation in both RNAs, and the green dots are genes with downregulated sRNA and upregulated mRNAs. **(D)** Validation of RNA-seq data by RT-qPCR. Gene expression was evaluated in WT spores interacting with macrophages versus saprophytic growth (left panel), as well as in the *r3b2*∆ mutant under the same conditions (right panel). Target genes included a permease, three transcription factors (*prd*, *box*, and *brca1*), and an LTR-transposable element. Data represent mean ± SD of three biological replicates, with individual replicates shown as circles. Statistical significance was determined using an unpaired t-test with Welch’s correction, with significance levels indicated above comparisons: *p < 0.05, **p < 0.01, ***p < 0.001, ****p < 0.0001.

While mRNA analysis enables the detection of genes regulated by the NCRIP, it does not distinguish between direct degradation events and secondary regulatory effects. To address this, we performed parallel sRNA sequencing on the same samples. This analysis revealed that macrophage interaction induces a strong activation of RNAi pathways, with the *r3b2*Δ mutant showing a markedly higher number of degraded targets (*r3b2*Δ-M versus *r3b2*Δ-C) than the WT strain (WT-M versus WT-C) (Fig 3A; S2 File). The accumulation of sRNAs in the mutant indicates that, in the absence of R3B2, the canonical RNAi pathway becomes hyperactive, suggesting that the NCRIP normally acts as a negative regulator of this process.

Additionally, we observed that NCRIP-dependent degradation was slightly more prominent during saprophytic growth (*r3b2Δ*-C versus WT-C) than during phagocytosis (*r3b2Δ*-M versus WT-M). Within these datasets, the majority of differentially expressed sRNAs showed lower abundance in the *r3b2*Δ mutant compared to the WT strain, both in the infection context (*r3b2*Δ-M versus WT-M) and under saprophytic conditions (*r3b2*Δ-C versus WT-C), indicating that their production largely depends on an active NCRIP (Fig 3A; S2 File). Together, these results demonstrate that NCRIP is a key regulator of RNA degradation both in basal conditions and during host interaction, reinforcing its role in fine-tuning gene expression relevant to pathogenicity.

Next, to determine the global correspondence between sRNA production and mRNA degradation, we plotted DEGs identified at mRNA level and annotated those showing a significant increase or decrease in sRNA accumulation (Fig 3B). Overall, a higher number of genes with significant changes in sRNA levels was detected in comparisons that also displayed a greater number of DEGs (Fig 3B). Furthermore, across all growth conditions, genes accumulating higher levels of sRNAs showed lower mRNA expression, whereas genes with reduced sRNA accumulation exhibited increased mRNA levels (S1A Fig).

This integrative analysis also allowed us to identify genes whose mRNA levels increase during macrophage interaction while sRNA production decreases, as these genes could play a crucial role in fungal pathogenesis by being selectively protected from degradation. The total number of genes exhibiting this behaviour was 24 in WT-M versus WT-C and 127 in *r3b2*∆-M versus *r3b2*∆-C (Fig 3C). In addition to its possible role during pathogenesis, genes that show a pattern (-sRNAs, + mRNA) in *r3b2*∆-M versus WT-M comparison were considered as direct targets of the R3B2 activity, this group includes 24 (Fig 3C), while in *r3b2*∆-C versus WT-C it comprises 5 potential target genes. Functional analysis revealed that most of these 24 potential R3B2 target genes are involved in signal transduction mechanisms, replication, nuclear structure, and chromatin structure organization. By contrast, the 127 genes from the *r3b2*∆-M versus *r3b2*∆-C and the 24 genes from WT-M versus WT-C are involved in metabolism and cellular processes, and signaling (S1B Fig).

### Fungal genetic markers of early phagocytosis and NCRIP regulation

To identify fungal genes that could serve as molecular markers of early phagocytosis or NCRIP regulation, we selected five candidate genes for transcriptional validation by RT-qPCR (Fig 3D). Selection of these genes was based on their consistent differential expression in both WT-M versus WT-C comparisons (Fig 3D**, left panel)** and *r3b2*Δ-M versus *r3b2*Δ-C (Fig 3D**, right panel)**, their relatively high expression levels in the RNA-seq data, and their predicted roles in stress adaptation, transcriptional regulation, or nutrient acquisition. The selected genes include: an LTR transposon (ID: 81899), which is a known direct target of the NCRIP in *M. lusitanicus* [19], a BRCT domain-containing protein (Brca1, ID: 113714; KOG4362), an HMG-box transcription factor (Box, ID: 104613; KOG2746), an amino acid transporter permease (ID: 165343; KOG1286), and the Prd transcription factor (ID: 83143; KOG0849).

The LTR transposon (ID: 81899) showed a distinctive expression profile. It was actively transcribed in the *r3b2*Δ mutant (*r3b2*Δ-C), whereas it remained transcriptionally silent in WT under saprophytic conditions (WT-C) (S2A Fig). Interestingly, in the *r3b2*Δ mutant, the LTR transposon decreased consistently during macrophage (~5.9-fold reduction in *r3b2*Δ-M versus *r3b2*Δ-C, p = 0.0059) (Figs 3D; S2A), indicating tight NCRIP control under non-stress conditions. By contrast, the BRCT domain-containing protein, HMG-box transcription factor, and amino acid transporter were significantly upregulated during early phagocytosis of both the mutant and WT strains. In WT spores, *brca1* increased ~1.9-fold (p = 0.0006) and *box* ~ 4.6-fold (p = 0.0003) compared to saprophytic conditions, while in the *r3b2*Δ mutant, *perm* showed a strong ~9.7-fold induction (p < 0.0001) and *box* increased ~2.5-fold (p = 0.0032). Conversely, the transcription factor *prd* was expressed at higher levels under saprophytic conditions (~2.5-fold, p = 0.0455), consistent with a role outside host–pathogen interaction. These quantitative data reinforce the notion that the permease and the transcription regulators Brca1 and Box might be key to host adaptation, while Prd may function primarily under non-infective growth conditions.

### Chronological transcription of chromatin and transcription-related genes during *M. lusitanicus* phagocytosis

**Fig 4:**
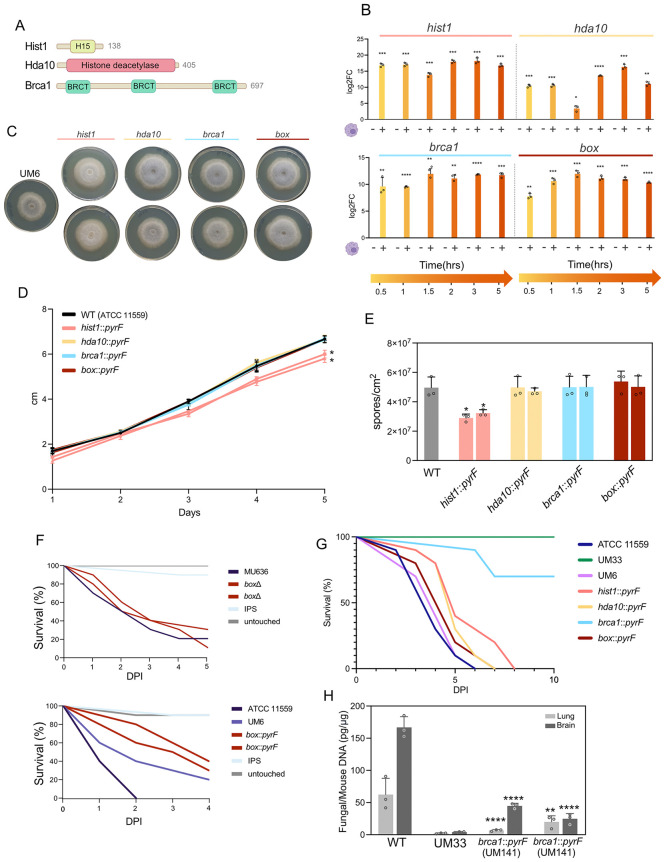
(A) Domain architecture of candidate genes encoding the transcriptional regulator Brca1, and the chromatin-associated proteins Hist1 and Hda10. (B) Temporal expression profile of *brca1*, *box*, *hist1*, and *hda10* in the WT strain (*M. lusitanicus*) during macrophage interaction (30 min-5 h post-infection) relative to saprophytic conditions. Data represent mean ± SD of 3 biological replicates; individual replicates are shown as circles. Statistical significance was determined using an unpaired t-test with Welch’s correction. Significance levels: *p < 0.05, **p < 0.01, ***p < 0.001, ****p < 0.0001. (C) Sporulation of the mutants with disruptions in transcriptional regulators and chromatin-related genes in *R. microsporus*. (D) Radial growth of the same mutants monitored every 24 hours over 5 days. (E) Spore production per cm² in the WT strain and each mutant after 2 days of growth. Data represent mean ± SD of three biological replicates; individual replicates are shown as circles. Statistical significance was assessed using one-way ANOVA followed by post hoc pairwise comparisons with Welch’s t-test. Significant differences are indicated by asterisks (* p < 0.05). (F) Survival plots of *G. mellonella* larvae infected with spores from WT and *box* mutant strains (upper panel, *M. lusitanicus*; lower panel, *R. microsporus*). Data represent the average of three independent experiments. (G) Survival plot of the murine model infected with spores of *R. microsporus* mutants disrupted in *brca1*, *box*, *hist1*, or *hda10*, along with virulent and avirulent control strains. Survival was analyzed using the log-rank (Mantel–Cox) test (p ≤ 0.05). DPI, days post-injection. (H) Quantification of fungal DNA in mouse lungs and brains after four days post-infection. Data mean ± SD of three biological replicates, with individual replicates shown as circles. Statistical significance was determined using one-way ANOVA. Significance levels: **p < 0.01, ****p < 0.0001.

To explore the contribution of RNA interference (RNAi) pathways and the impact of early macrophage phagocytosis on fungal gene regulation, we focused on four candidate genes showing differential expression at the small RNA (sRNA) and/or messenger RNA (mRNA) levels under various experimental conditions (WT-M, WT-C, *r3b2*∆-M, and *r3b2*∆-C). These genes included two chromatin-related proteins, Histone H1 (GeneID: 83400), a histone deacetylase (GeneID: 168144), and the two above-mentioned transcription regulators, Box (GeneID: 104613) and Brca1 (GeneID: 113714) (Fig 4A).

The genes encoding histone deacetylase and Brca1 exhibited a strong accumulation of both sRNA and mRNA in the *r3b2*∆ mutant during macrophage interaction (*r3b2*∆-M versus *r3b2*∆-C), suggesting regulation by the canonical RNAi pathway and additional induction by phagocytic stress (S2B Fig). In contrast, *box* and *his1* displayed expression changes mainly at the mRNA level, with minimal sRNA variation, implying RNAi-independent transcriptional regulation (S2B Fig).

Next, since phagocytosis of *M. lusitanicus* spores leads to transcriptional reprogramming, likely involving chromatin remodelling and transcriptional regulation necessary for survival and germination within the phagosome, we aimed to characterize these early transcriptional changes. To this end, total RNA was extracted from the WT strain at multiple time points (30 min, 60 min, 90 min, 2 h, 3 h, and 5 h), as well as from L15 medium as a non-infection control. At 1 h post-infection, RT-qPCR analysis revealed significant induction of the chromatin-related genes: *hist1* increased ~17-fold compared to saprophytic conditions (p = 0.0003), and the histone deacetylase *hda10* increased ~10-fold (p = 0.0006). Similarly, the transcriptional regulators *brca1* and *box* were upregulated by ~9-fold (p < 0.0001) and ~11-fold (p = 0.0009), respectively (Fig 4B). Gene induction patterns remained broadly consistent across the other time points, indicating a sustained activation of chromatin- and transcription-associated genes throughout phagocytosis. These quantitative changes illustrate that even at an early stage of phagocytosis, genes involved in chromatin remodeling and transcriptional regulation are strongly induced, suggesting they may play a central role in orchestrating virulence-associated transcriptional programs.

### Evolutionary conservation of the RNAi-regulated and phagocytosis marker genes

To understand the evolutionary conservation of the RNAi-regulated and phagocytosis marker genes in Mucorales, we analyzed their presence across 61 fungal species representing major phyla. We extended the comparison to metazoans, including *Mus musculus*, *Homo sapiens*, and *Drosophila melanogaster.*

Starting with encoding histone deacetylases (HDAs), we identified seven and six copies in *M. lusitanicus* and *R. microsporus*, respectively. Among them, we found the *hda* gene studied in this work, which is a putative ortholog of human HDA10, supporting its annotation as *hda10* (S4A Fig). Similarly, in other fungal species, copy numbers ranged from 6–10 HDA genes, while metazoans displayed higher numbers, suggesting lineage-specific expansions. Building on the analysis of HDAs, we next examined Histone H1, a linker histone that plays a key role in chromatin compaction [20]. Interestingly, both *M. lusitanicus* and *R. microsporus* harbor a single histone H1 gene, whereas other fungal species contain 1–6 copies, and some, such as *P. blakesleeanus*, lack detectable homologs (S4B Fig).

Having characterized the evolutionary conservation of chromatin-related genes, we next examined additional transcriptional regulators, focusing on BRCT-domain and box-domain proteins. BRCT-domain proteins, which participate in BRCA1-like DNA repair, transcriptional regulation, and cell cycle control [21], displayed substantial variation across species. The number of detected BRCT-domain proteins ranged from zero in some fungi (*H. vesiculosa*, *U. maydis*) to 32 in humans, reflecting extensive gene duplication and loss across evolution (S4B Fig). Consistent with this variability, *M. lusitanicus* and *R. microsporus* contain nine and seven copies, respectively. This contrast between fungal and metazoan distributions was also evident for box-domain proteins, which were detected exclusively in fungi (S4B Fig). Within Mucorales, *M. lusitanicus* harbors two box-domain proteins, whereas *R. microsporus* contains a single copy.

### Disruption of the chromatin and transcription-related genes impairs *R. microsporus* virulence and fungal fitness

To assess their role in virulence and fungal development, and to elucidate the interplay between canonical and non-canonical RNAi pathways in the context of host-pathogen interactions, we generated single deletion mutants in *M. lusitanicus* (S3A Fig). To evaluate whether these genes perform conserved functions across species, the corresponding orthologs in *R. microsporus* were also disrupted (S3B Fig).

First, the mutant strains were phenotypically characterized by monitoring their sporulation (Figs 4C, E; S4C, S4E) and vegetative growth (Figs 4D; S4D). In *R. microsporus*, both *hist1* independent mutants (UM145 and UM146) showed a clear sporulation defect, producing ~40% fewer spores than the WT strain (Fig 4C, E). These mutants also displayed reduced radial growth, with colony diameters consistently ~10–15% smaller than WT across the five-day assay (Fig 4D). No growth or sporulation defects were observed for the *hist1Δ* mutant in *M. lusitanicus*, nor for any of the other mutants in either species. Statistical analyses for all phenotypic measurements are summarized in S1 Table.

We next evaluated the effect of temperature and light on mutant growth. Under darkness at 37 °C, only the *brca1* independent mutants of *R. microsporus* exhibited a phenotype, showing a striking increase in sporulation, approximately 9- to 13-fold relative to WT (S5 Fig). This observation suggests that disruption of this transcription factor triggers a temperature-dependent stress response. Statistical tests for these experiments are also reported in S1 Table.

Subsequently, a first screening of their impact on pathogenicity was performed using the *Galleria mellonella* infection model (Figs 4F; S6A, S6B), allowing us to evaluate their contribution to virulence in both species. *G. mellonella* larvae were challenged with the *M. lusitanicus* and *R. microsporus* strains (S2 Table).

In *M. lusitanicus*, deletion of any of these genes (S6A Fig), including the conserved transcription factor *box* (Fig 4F**, upper panel),** had no significant impact on virulence. Independent mutants for each locus displayed survival curves comparable to the control strain (median survival 48–72 h; Log-rank p > 0.2 in all cases), indicating that loss of these chromatin modifiers or transcriptional regulators does not measurably affect pathogenicity in this species.

In contrast, *R. microsporus* mutants exhibited gene-specific and robust virulence phenotypes. Disruption of the transcriptional regulators *brca1* (*brca1::pyrF*) and *box* (*box::pyrF*) led to marked attenuation of virulence in the *G. mellonella* infection model. The *brca1::pyrF* mutants (UM141 and UM142) were almost avirulent (median survival undefined vs. 24 h in the wild type; p < 0.0001) (S6B Fig), while *box::pyrF* mutants (UM143 and UM144) showed prolonged host survival (84–96 h; p = 0.0005–0.0001) (Fig 4E**, lower panel)**.

Among the chromatin-related genes, disruption of *hda10* (*hda10::pyrF*) strongly reduced virulence in one independent mutant (UM121; p = 0.0001) but not in the second (UM122; p = 0.44). Loss of function of *hist1* (*hist1::pyrF*, UM145) produced a moderate, though not statistically significant, increase in survival (median 84 h; p = 0.092), suggesting a potential contribution to virulence regulation (S6B Fig).

To further validate these findings in a mammalian host, the *R. microsporus* mutants were tested in a murine infection model. The *brca1::pyrF* mutants, which had the strongest attenuation in *G. mellonella*, were both severely attenuated in mice as well (p < 0.0001) (Figs 4G, S6C), confirming Brca1 as a critical virulence determinant. The chromatin-related mutants *hist1::pyrF* (UM145; p = 0.0204) and *hda10::pyrF* (UM121; p = 0.0372) also showed significant reductions in pathogenicity, whereas the *box::pyrF* mutant (UM143; p = 0.23) displayed no detectable effect in mice (Fig 4G).

Consistent with these findings, we assessed fungal burden and spore survival to investigate the attenuated virulence of the *brca1* mutants. Fungal DNA was quantified in lung and brain tissues of infected mice four days post-infection. Both *brca1* mutants (UM141 and UM142) showed significantly lower fungal burden in lungs and brain compared to the wild-type strain, with levels comparable to the avirulent control UM33 (UM141: lung p < 0.0001, brain p < 0.0001; UM142: lung p = 0.0023, brain p < 0.0001) (Fig 4H). This additional *in vivo* fungal burden analysis provides an independent validation of the reduced virulence phenotype observed for the *brca1* mutants.

In parallel, the survival of spores from the virulent (ATCC 11559), the avirulent (UM33), and the two *brca1* mutant strains after 24 h interaction with peritoneal immune cells was evaluated. Spores were recovered from the peritoneal fluid and plated alongside non-injected controls to calculate survival rates. Both *brca1* mutants exhibited significantly reduced survival compared to wild-type spores (UM141: p = 0.009; UM142: p = 0.0033; UM33: p < 0.0001) (S6D Fig), indicating that their attenuated virulence is associated with diminished fitness during host immune interaction.

## Discussion

In response to fungal invasion, macrophages act as the first line of defense, recognizing and internalizing Mucoralean spores to initiate phagosome maturation. This process exposes the spores to acidic pH, reactive oxygen species, and nutrient deprivation, aiming to eliminate them [12]. However, depending on the fungal species and the immune status of the host, some spores can survive, germinate, and escape, leading to infection [12]. While previous studies have shed light on the later stages of infection [18], the genetic regulation underlying the early interaction between *M. lusitanicus* and macrophages remains largely unexplored. To address this gap, we selected *M. lusitanicus* as a model organism due to its genetic tractability and included *R. microsporus*, a major causative agent of mucormycosis, due to recent advancements in its genetic manipulation [22]*.* This dual-species approach enables the identification of conserved virulence factors and situates the early transcriptional response within the broader context of Mucorales pathogenesis.

Our transcriptomic analysis revealed extensive early transcriptional reprogramming upon macrophage phagocytosis, with upregulation of genes involved in amino acid transport, energy metabolism, and translation, suggesting immediate metabolic adaptation to phagosome stress. This metabolic activation aligns with findings from previous studies on the late stages of *M. lusitanicus* phagocytosis [23]. This early response exceeds transcriptional changes observed at later stages and is consistent with findings in other fungal pathogens, including *Candida* species, where early macrophage interaction similarly elicits strong transcriptional changes [24–26]. While comparable controls were used for each time point, it should be taken into account that part of these differences may be due to variations in gene expression related to morphogenesis, since the presence of macrophages can alter germination rates. Overall, these data highlight the dynamic fungal response to host immunity and the critical role of early transcriptional shifts in survival and pathogenesis.

In our study, we integrated the analyses of sRNA and mRNA profiles during the early phagocytosis of *M. lusitanicus* spores by macrophages to elucidate the regulatory networks involved. This integrated degradome approach revealed that both the NCRIP and the canonical RNAi pathways coordinate gene regulation in response to phagocytosis, thereby promoting stress adaptation and intracellular survival [18,27].

**Fig 5:**
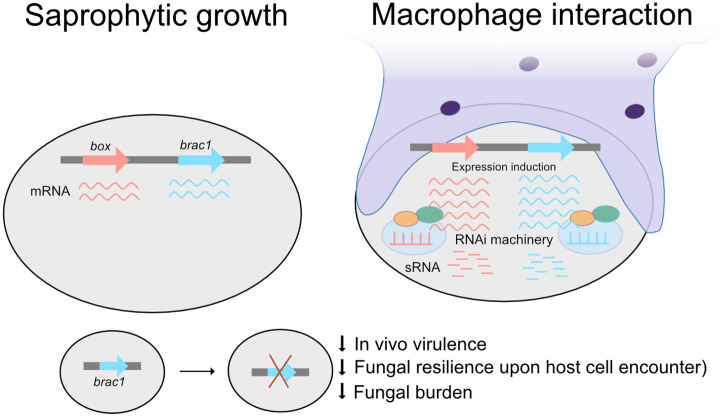
Schematic representation of the regulation of genes induced during the interaction with macrophages and targeted by RNAi. Below, a schematic depiction of the *brca1* gene disruption in *R. microsporus* and its effects on pathogenic capacity is shown.

During phagocytosis, *M. lusitanicus* spores activate a coordinated network of chromatin remodeling and transcriptional reprogramming that enables survival and germination within the hostile environment of the host phagosome. Chromatin-related proteins such as Histone H1 and Hda10, together with transcription factors including BRCA1 and Box, appear to act as central regulators of fungal adaptation, integrating multiple layers of gene regulation to rapidly respond to host-imposed stresses. Notably, the differential regulation of these genes, some modulated by RNAi, others primarily through transcriptional mechanisms, suggests a modular strategy whereby Mucorales orchestrate early transcriptional responses critical for infection (Fig 5). Building on this regulatory framework, the functional analyses of Brca1, Histone H1, Hda10, and Box provide evidence that chromatin and transcriptional control are directly linked to virulence potential (Fig 5).

The reduced virulence, lower fungal burden, and impaired survival of the *brca1* mutant support that genome stability and DNA repair are key determinants of fungal fitness under immune pressure [21]. This central role of the Brca1 protein is further highlighted by phylogenomic analysis, which revealed variability in BRCA1-domain proteins across species, indicating potential evolutionary divergence driven by host interaction or environmental adaptation. This core function of Brca1 is not isolated but might be integrated within a broader network of chromatin-related proteins such as Histone H1 and Hda10, which were also critical for virulence, suggesting a coordinated adaptive strategy. The Histone H1, although non-essential for viability in organisms such as *Saccharomyces cerevisiae* [28], *Tetrahymena thermophila* [29], *Neurospora crassa* [30], and *Aspergillus nidulans* [31], its functional role in chromatin regulation is context-dependent [29,30]. Consistently, in our study, *hist1* disruption impaired virulence in *R. microsporus*, while effects in *M. lusitanicus* were more modest. This observation highlights conserved yet species-specific roles of H1 in chromatin-mediated regulation during host interaction, emphasizing how Histone H1 potentially may collaborate with Brca1 to orchestrate the transcriptional responses crucial for survival under immune pressure.

Similarly, Hda10 supports this virulence regulatory network. This protein has been implicated in regulating virulence-related pathways, including morphogenesis, stress response, and drug resistance, highlighting its potential as an antifungal target, in human fungal pathogens such as *Candida albicans*, *C. glabrata*, and *Cryptococcus neoformans* [32,33]. Our findings support a similar role in Mucorales, where Hda10 contributes to fungal pathogenicity in *R. microsporus*, likely through chromatin-mediated transcriptional responses activated during host interaction.

Finally, the transcription factor Box also contributes to this virulence-associated regulatory circuitry. It showed reduced virulence in *R. microsporus* in *G. mellonella* but not in the murine model, underscoring species-specific contributions. Notably, its conservation among fungi, coupled with its absence in broader eukaryotes point to a specialized role in fungal pathogenesis, making Box, alongside Brca1, a compelling candidate for antifungal targeting.

This virulent regulatory network headed by Brca1 could be further explored in future studies through the interaction between these proteins and their downstream targets. Our understanding of this network began to take shape through host–pathogen interaction studies in *M. lusitanicus*, a model that has provided valuable initial insights into the molecular players involved. Notably, mutants in *M. lusitanicus* did not always exhibit strong virulence defects, likely because this species mostly retains pathogenic potential in immunocompromised hosts [18], combined with the general functional redundancy between paralogous genes that surged after an ancestral genome duplication in the clade [34]. These findings underscore that different Mucorales species likely harbor distinct virulence determinants, suggesting that a combination of model systems is essential for a comprehensive understanding of the molecular mechanisms driving fungal infections.

In the present study, we also established a novel method to assess fungal survival via direct interaction with immune cells extracted from the peritoneum, followed by *in vitro* recovery and plating of spores. This approach complemented results obtained from retroorbital infection and fungal burden assays, further highlighting the critical role of Brca1 in *R. microsporus* pathogenesis. Future studies employing physiologically relevant *in vivo* models and diverse host environments will be essential to validate the regulatory networks uncovered here, and to translate these insights into potential strategies for antifungal intervention.

## Methods

### Ethics statement

The virulence assays in this work were performed according to the ethical Guidelines of the European Council (Directive 2010/63/EU) and the Spanish RD 53/2013, ensuring animal welfare, by minimising any potential pain, distress, or suffering experienced by the mice along the course of the experiments. Procedures and experiments conducted in this study were closely supervised and granted approval by the University of Murcia Animal Welfare and Ethics Committee, as well as the Council of Water, Agriculture, Farming, Fishing and Environment of Murcia, Spain (authorization number REGA ES300305440012).

### Fungal strains and culture conditions

The *M. lusitanicus* strains MU636 (*leuA*^*-*^) and MU412 (*leuA, r3b2*-^-^) were used in the host-pathogen in vitro interaction. Both strains have the same genetic background [17,35]. MU636 was employed as a recipient strain for the deletion of the transcription regulators *brca1* and *box* and the chromatin-related genes *hist1* and *hda10*. Also, together with R7B, MU636 was used in the *G. mellonella* infection. On the other hand, the NRRL3631 was the avirulent control.

For optimal growth and sporulation, *M. lusitanicus* strains were grown in rich medium (YPG; 3 g/L yeast extract, 10 g/L peptone, 20 g/L glucose, 15 g/L agar) pH 4.5 at 26 ºC and under illumination. Subsequently, spores were harvested, washed, and filtered using a 70 μm strainer before the host-pathogen interaction.

All the *R. microsporus* strains used in this work derived from the WT *Rhizopus microsporus* ATCC 11559 (listed in S2 Table), which was used in the mouse infection as the virulent control. UM6 was also used as a virulent strain. The avirulent control was the UM33, which was employed as the receptor strain for the mutant generation. All *R. microsporus* strains were cultured in rich medium YPG pH 4.5 at 30ºC for spore harvesting, supplemented with uridine (200 mg/l) when required. All the media and culture conditions used for *M. lusitanicus* and *R. microsporus* growth are detailed in Lax et al. [22].

### *In vitro* host-pathogen interactions

The *in vitro* interaction of MU412 and MU636 spores with macrophages was conducted following the procedure described in Pérez-Arques et al. [23]. Briefly, *M. lusitanicus* spores were confronted with the mouse cell line of macrophages J774A.1; ATCCTIB-67 in a proportion of 1.5:1 (spores: macrophages). For the RNA-seq analysis, spores were exposed to macrophages for 1 h to conduct early phagocytosis. For the chronological expression of the gene candidates involved in the early phagocytosis for the RT-qPCR analysis, confrontations of MU412 and MU636 spores with macrophages were performed for 30 min, 60 min, 90 min, 2 h, 3 h, and 5 h. The in vitro interaction with macrophages was performed at 37ºC in an L15 medium (Capricorn Scientific) supplemented with Fetal Bovine Serum (FBS, Capricorn Scientific). For the saprophytic conditions, the same concentration of MU412 and MU636 spores was cultured in L15 supplemented with FBS. For the Lysotracker Green DND-26 (ThermoFisher) and DAPI (Life Technologies) staining, the protocol described in [15] was followed.

### RNA extraction, library preparation, and RNA-seq analysis

For the *in vitro* host-pathogen interactions, total RNA was extracted from three replicates of MU636 and MU412 strains interacting with macrophages (after 1 h) and three replicates of each of these strains growing for 1 h in saprophytic conditions. Total RNA (sRNA + mRNA) was purified from the 12 samples using the MirVana Kit (Invitrogen) and following the procedure for sRNA enrichment. Each sample was divided into two, one for mRNA sequencing and the other for sRNA sequencing. Samples were quality-checked, quantified, and deeply sequenced by Novogene.

For the *in vivo* infection, three replicates from the host-pathogen interaction were used for total RNA extraction using the mirVana kit. Additionally, three replicates of the fungal spores growing in saprophytic conditions, as well as three replicates from the mouse cell samples (in a non-infection context) were also extracted and deeply sequenced by the same company. Raw sRNA-seq reads were quality-checked with FASTQC v0.11.8 (https://www.bioinformatics.babraham.ac.uk/projects/fastqc/) before and after removing adapter (3´) and contaminant sequences and low-quality reads (-q28-p50) were removed with Cutadapt v3.4 (https://cutadapt.readthedocs.io/en/v3.4/). Clean reads between 18 and 35 nt were aligned to the *M. lusitanicus* v2.0 genome (retrieved from https://mycocosm.jgi.doe.gov/Mucci2/Mucci2.home.html) [36] using Bowtie2 v2.5.3 [37]. Reads mapping on the *M. lusitanicus* genome were analysed together with FeatureCounts v2.0.1 [38]. The software produced a count table file (with several reads from each library that defined each locus), which was used for differential expression (DE) analysis between the mutant and wild-type strains (with or without macrophages for the in vitro host-pathogen interaction) with DESeq2 v2.11.40.6 [39]. Genes with an adjusted p-value ≤0.05 and a log2 fold-change |(log2FC) ≥1| were considered as differentially expressed.

Raw mRNA datasets were checked for quality with FASTQC before and after removing adapter and contaminant sequences with TrimGalore! v0.6.2 (https://github.com/FelixKrueger/TrimGalore) excluding reads with a Phred quality score Q ≤ 32 and/or a total length ≤20nt as well as adapter sequences with an overlap ≥4 bases. The mRNA clean reads were aligned to the *M. lusitanicus* v2.0 genome using HISAT2 aligner [40]. HTSeq software v.2.0.5 [41] was used to quantify gene expression levels, and differential expression analysis was performed using DESeq2, considering genes with an FDR ≤ 0.05 and |log2FC| ≥ 1 as significantly differentially expressed.

KOG class enrichment analyses for *M. lusitanicus* DEGs were performed in GraphPad Prism v8.0.1; delta-ranks were computed as the difference between the mean of all genes within the KOG class and the mean rank of all other genes in a Mann-Whitney U-test. A KOG class was considered over-represented if P ≤ 0.05 in one-sided Fisher´s exact evaluation (performed in R) of the DEGs compared to the total amount of genes in each KOG class.

To assess the chronological dynamics of gene expression in the WT strain during phagocytosis, differential expression analyses were performed independently for each timepoint (1 h and 5 h) by comparing macrophage-interacting spores to their respective saprophytic controls. Subsequently, genes consistently differentially expressed across both timepoints, transiently regulated at one timepoint, or oppositely regulated between early (1 h) and late (5 h) phagocytosis were identified to characterize temporal transcriptional patterns, without pre-assuming the underlying biological processes.

### RT-qPCR quantification

Once total RNA was extracted, about 5 μg of total RNA of *M. lusitanicus* WT (MU636) and mutant (MU412) strains samples were treated with the Turbo DNase (Thermo Fisher). The RNA samples were routinely checked for DNA contamination by a PCR analysis using primers for the housekeeping elongation factor gene. For cDNA synthesis, 1 μg of total RNA was reverse-transcribed using the iScript cDNA synthesis kit (Bio-rad) at 25 ºC for 10 min, 42 ºC for 50 min, and 70 ºC for 15 min. The RT-qPCR was performed in triplicate using 5X SYBER green PCR master mix (Applied Biosystems) with a QuantStudio 5 flex system (Applied Biosystems) following the supplier´s recommendations. To ensure non-specific amplification, non-template control and melting curves were tested. The primer sequences used for the quantification of genes are listed in S3 Table. The efficiencies of every pair of primers were approximately identical, and the relative expression of target genes was obtained by the delta-delta cycle threshold (ΔΔCt) method, normalizing for the endogenous control elongation factor gene (*ef*).

### Nucleic acid manipulation and plasmid constructions

All PCR amplifications were conducted using the Herculase II fusion DNA polymerase (Agilent, Santa Clara, California, USA), with annealing temperature and extension time adjusted based on the specific pair of primers used and the fragment length to be amplified, respectively. PCR fragments were purified from gel when required using the GeneJet Gel Extraction kit (Thermo Scientific). DNA extraction was conducted following the previously described protocol in [41]. All primers were designed using the Primer3Stat (https://www.bioinformatics.org/sms2/pcr_primer_stats.html) and Multiple Primer Analyzer (https://www.thermofisher.com/es/es/home/brands/thermo-scientific/molecular-biology/molecular-biology-learning-center/molecular-biology-resource-library/thermo-scientific-web-tools/multiple-primer-analyzer.html) tools.

### Deletion cassettes for phagocytosis-related mutants in *M. lusitanicus*

The phagocytosis-related single mutants were achieved using genetic constructions consisting of the selectable marker *leuA* flanked by 1 Kb upstream and downstream of the gene candidates. The 1 Kb upstream, the 1 Kb downstream, and the selectable marker were PCR amplified using specific primers (S3 Table). Subsequently, by overlap PCR the three fragments were fused generating the deletion fragment. The deletion cassettes were purified and used for the protoplast transformation of the MU636 *M. lusitanicus* strain. Transformants were acquired following the previous transformation procedure [42]. In short, harvested fresh spores were incubated for 2–4 hours ensuring their correct germination. Subsequently, protoplasts were obtained by cell wall digestion of the germinated spores with lysing enzymes (Sigma-Aldrich) and chitosanase (Sigma-Aldrich). The protoplast transformation was performed by electroporation with *SaclI* linearized plasmids and later incubated in poor agar medium YNB + Sorbitol 3.2 and checked 3–4 days for colonies. Approximately, 5 μg of overlapping PCR products, which include the *leuA* gene flanked by 1 Kb upstream and downstream the open reading frame (ORF) of each of the candidate genes, was necessary for each transformation. As spores are multinucleated, colonies were subsequently transferred to fresh YNB agar plates (5–10 vegetative cycles) to obtain homokaryonts with all nuclei transformed. DNA from transformants was extracted as previously described. Homokaryosis was checked using primers that bind upstream and downstream the 1Kb employed for the fusion PCR to ensure the absence of WT nuclei (S4 Fig).

### Disruption cassettes for phagocytosis genes in *R. microsporus*

Disruption of the orthologous genes in *R. microsporus* (*hist1* [ID: 1913394], *hda10* [ID: 1870091], *brca1* [ID: 1897364], and *box* [ID: 1900053]) was performed using the CRISPR-Cas9 system, following the genetic disruption protocol described by Lax et al. [43]. Briefly, crRNAs (S4 Table) were designed using the EuPaGDT gcRNA design tool (http://grna.ctegd.uga.edu/) with default parameters to guide the Cas9 cut. For homology-directed repair, primers amplifying the *pyrF* selective marker were designed with 38 bp of homology upstream and downstream of the cut point. To verify integration, a reverse primer specific to *pyrF* and a forward primer that hybridizes upstream of the cut site were designed. Homokaryon checking was performed using the forward upstream primer and a reverse downstream primer to the cut site. All primers were designed using Primer3Stat and the Multiple Primer Analyzer tools.

For the transformation of *R. microsporus*, the UM33 recipient strain (*pyrF-*, *LeuA-*) was used. Protoplasts of UM33 were transformed by electroporation with 150–200 mg/ml of linear DNA fragment (*pyrF* flanked by 38 bp tails). Transformed protoplasts were then cultured on MMC selective media (without uridine). Genomic DNA of the transformants was extracted following the procedure outlined by Osorio-Concepcion et al. [35] for integration and homokaryon checking.

### Phenotypic characterization of the phagocytosis mutants

Radial growth and sporulation of the phagocytosis mutants were analysed, inoculating drops of 500 spores in the plate center of MMC 3.2 (for radial growth) and YPG 4.5 (for sporulation). Radial growth was monitored during 5 d each 24 h through measuring the colony diameter. Spore production was determined by quantifying the spore concentration of 1 cm^2^ of a chunk of agar, which was later transferred to a 50 ml falcon containing 10 ml of PBS1X. Subsequently, the falcon was vigorously vortexed to enable spore release, which were counted using a Neubauer camera, determining the spore concentration of the mutant and the WT strains. Both radial growth and spore production were quantified using three biological replicates per mutant, and all measurements are reported as mean ± SD. Statistical analysis was conducted using one-way ANOVA followed by Welch’s t-test, considering differences statistically significant at p*≤* 0.05.

### Virulence assays

To determine if the phagocytosis-related genes constitute conserved virulence factors in Mucorales, as a first screening, *G. mellonella* larvae were infected with *M. lusitanicus* mutants and the corresponding ortholog mutants in *R. microsporus.* To this end, sixth instar larvae of *G. mellonella* (SAGIP, Italy), weighing 0.3 -0.4 g, were selected for experimental use. Larvae, in groups of twenty, were injected through the last pro-leg into the hemocoel with 106 spores in a volume of 20 μl according to Kelly & Kavanagh [44] and incubated at 37 °C. Untouched larvae and larvae injected with sterile insect physiological saline (IPS) served as controls. Survival was monitored every 24 h for the duration of 96 h. Experiments were repeated at least 3 times.

On the other hand, to assess the role of phagocytosis-related genes in *R. microsporus* virulence, survival assays were conducted as previously described by Lax et al. [22]. *M. lusitanicus* mutants were discarded from the mice infection, due to the absence of significant reduction in virulence obtained in *G. mellonella.* Briefly, healthy Swiss mice weighing ≥30 g and one month old (supplied by the Animal Facility Services, University of Murcia, Spain) were used as the host model for virulence assays. Groups of 10 mice (per strain) were injected via retroorbital infection with suspensions of 1x10^6^ from the mutant or the WT strain. Mice were housed under established conditions with free food access and sterile water. The survival rates of each group were checked twice a day for 10 d, and mice reaching the endpoint were euthanized in a CO_2_ chamber. Both in *G. mellonella* and in mice virulence assays, statical analysis was performed by Mantel–Cox test, and differences in survival were considered significant with a P value ≤ 0.05. To evaluate spore survival after interaction with immune cells we injected spores (1x10^6^) into the peritoneum of mice using the two independent *brca1* mutants (UM141 and UM142) as well as the control strains (UM33 and ATCC 11559). After 24 hours, we extracted the peritoneal fluid after injecting 5 ml of L15 medium. A fixed number of spores were plated (100 per plate). In parallel, we plated the same number of spores that had not been exposed to the peritoneum. We then assessed the survival of these spores after interaction with the immune cells present in the peritoneal cavity and observed that the survival of spores by counting individual colonies. For fungal burden analyses, organs were ground up on liquid nitrogen and gDNA was extracted as previously described [17,43]. For DNA quantification by real-time PCR (qRT-PCR) specific primers of *R. microsporus* chitin synthase gene (ID1911606) and mice β2 microglobulin gene (ID12010) were used (S3 Table). Sample analyses were carried out in triplicate in 10 µl PCR reactions containing 150 ng of test sample gDNA from three individuals using SybrGreen kit (Fast SYBR Green Master Mix – Agilent) from non-infected mice was used as negative control. Relative amount of fungal and mouse gDNA was quantified on the basis of their standard curves, elaborated with known fungal DNA concentrations (0.005 ng–10 ng) in a background of 150 ng of non-infected mice gDNA and mouse DNA concentrations (1 ng–200 ng) and their corresponding amplification cycle threshold (Ct).

### Phylogenetic analysis and ortholog search

Proteomes of 64 representative species were retrieved from the Joint Genome Institute (JGI) MycoCosm genome portal [45] and Uniprot [46]. Sequences of *M. lusitanicus* Histone 1 (ID: 83400), Hda10 (ID: 168144), Brca1 (ID:113714), Box (ID: 104613) proteins were queried against the selected proteomes using iterative HMMER jackhmmer searches (E-value ≤ 1x10-3) (v3.3.2) (http://hmmer.org/). A reciprocal BLASTp search (v2.10.1) [47] was conducted and sequences that failed to produce a hit were discarded. An additional search using Pfam-A database [48] using HMMER hmmscan (v3.3.2) (http://hmmer.org) served to remove hits that lacked the BRCT domain and BRCA1-associated (Brca1 search), the Histone deacetylase domain (HDA search), or the Linker histone H1/H5, domain H15 (Histone 1 search) (S3 File). For the Box search, domain-based filtering was not applied, since most Box orthologs identified in fungi do not contain any known conserved domains. The remaining Histone 1, Hda10, Brca1, and Box candidate sequences were used to determine the copy number of each protein type across all analyzed species. This data was compiled into a matrix representing the number of copies per species, which was subsequently used to generate a heatmap illustrating the distribution and expansion of each protein family across fungal lineages. The Hda candidate sequences were further aligned using Clustal Omega [49] with default parameters. A phylogenetic tree was then generated from this alignment using Clustal Omega’s built-in approximate maximum-likelihood method with default settings. The species tree was generated after analyzing the 64 proteomes with OrthoFinder (Inflation factor, -I 1.5) [50]. Phylogenetic trees were visualized in iTOL [51].

### Code availability

The scripts used for the bioinformatic analysis are available at https://github.com/ghizlanetahiri95/Early_Phagocytosis_Mucorales_RNAi.

## Supporting information

S1 Fig (A) Correlation between sRNA production and mRNA expression levels. The top 100 genes with the highest and lowest sRNA production were selected for the WT strain with and without macrophages, and the *r3b2*Δ with and without macrophages. The mRNA expression levels of these top 100 genes, represented as the log2 of FPKM, were plotted for each group. Boxplots indicate the median, first, and third quartiles; whiskers extend to the 10th and 90th percentiles. Statistical significance was assessed using Welch’s t-test. Significance levels: *p < 0.05, ***p < 0.001. **(B)** Functional analysis of genes showing increased mRNA levels and decreased corresponding sRNAs during interactions of WT and *r3b2*∆ strains with macrophages across the comparisons: WT-M/ WT-C, *r3b2∆-*M/ WT-M, and *r3b2∆-*M/*r3b2∆-*C).
(TIFF)

S2 Fig (A) Genomic coverage of sRNA and mRNA reads mapped to the LTR-transposon and adjacent genes in WT and *r3b2*∆ strains, under the same conditions. **(B)** Genomic coverage of sRNA and mRNA reads mapped to the transcription factors *brca1* and *box*, and the chromatin-associated genes *hist1* and *hda10* in WT and *r3b2*∆ strains, under saprophytic and macrophage-interacting conditions. Yellow and red plots indicate sRNA and mRNA read coverage, respectively.
(TIFF)

S3 Fig (A) Homokaryosis PCRs of the mutants in the potential virulence factors in *M. lusitanicus.* PCRs to check if the mutants are homokaryonts were conducted using a forward and a reverse primers that bind upstream and downstream the regions used for the fusion (Locus_F and Locus_R). The PCR products in the deleted mutant result in 5 Kb, 5 Kb, 5.1 Kb, and 5.3 Kb in the *hist1, brca1, hda10, and box* loci respectively. The PCR products with the wild-type genes have 0.63 Kb, 4.8 Kb, 3.5 Kb, and 4.3 Kb for *hist1, brca1, hda10*, respectively. Red boxes show the expected PCR products in the deletion nuclei. The heterokaryons were submitted to further vegetative cycles in selective media. PCRs to check homokaryosis of *R. microsporus* mutants in phagocytosis-related genes**. (B)** PCRs to check *R. microsporus* homokaryosis of the gene candidate disruptions, using specific primers that hybrid approximately 1 Kb from the 38 bp homology regions. The amplification fragments lengths (shown in red boxes) of the disrupted genes are 5.5Kb for *hist1, hda10, and brca1* disruptions, and 5.3 Kb for the *box* disruption. The amplification product lengths resulting from the WT nuclei are 2 Kb for the *hist1, hda10, and brca1* genes, and 1.8Kb for the *box* locus. The mutants containing only nuclei with the disruptions (red rows) were selected for the subsequent analysis.
(TIFF)

S4 Fig (A) Phylogenetic analysis of HDA homologs. Maximum likelihood phylogeny of histone deacetylase (HDA) proteins from *M. lusitanicus* (red), *R. microsporus* (violet), and other representative species from the Early Diverging Fungi (EDF), Ascomycota, Basidiomycota, and Metazoa. Human HDA isoforms were included as outgroups to classify fungal HDAs and infer potential functional conservation. **(B)** Comparative phylogenomic distribution analysis showing the presence/absence and copy number of Brca1, Box, HDA10, and Histone H1 homologs across multiple fungal genomes. *M. lusitanicus*, *R. microsporus* are indicated by red and violet dots, respectively, and other opportunistic human pathogens with blue dots. **(C)** Sporulation of mutants with deletions in transcriptional regulators and chromatin-related genes in *M. lusitanicus*. **(D)** Monitoring of radial growth of the same mutants every 24 hours over 5 days. **(E)** Spore production per cm² in the WT strain and each mutant after 2 days of growth. Statistical significance was assessed using one-way ANOVA followed by post hoc pairwise comparisons with Welch’s t-test. No asterisks indicate that differences were not statistically significant.
(TIFF)

S5 Fig Effects of temperature and light on mutant sporulation. **(A)** Sporulation of two *R. microsporus* mutants disrupted in the *brca1* gene was evaluated under dark conditions at 37°C. **(B)** Plates showing sporulation differences between the mutants and WT strain at 37ºC and in darkness. Statistical analysis was assessed using one-way ANOVA followed by post hoc pairwise comparisons with Welch’s t-test. Significant differences are indicated by asterisks (* p < 0.05, ** p < 0.01).
(TIFF)

S6 Fig Virulence and survival assays of *hist1*, *hda10*, and *brca1* mutants. **(A)** Average survival of *G. mellonella* larvae infected with *M. lusitanicus* spores from the indicated mutants and the WT strain. Data represent the mean of three independent experiments. **(B)** Average survival of *G. mellonella* larvae infected with *R. microsporus* spores from the corresponding mutants. **(C)** Survival of mice infected with *R. microsporus* spores from two independent *brca1* mutants disrupted in *brca1*, along with virulent and avirulent control strains. Survival was analyzed using the log-rank (Mantel–Cox) test (*p* ≤ 0.05). DPI, days post-injection. **(D)** Survival rate of spores from WT, avirulent strain (UM33), and *brca1* mutant strains (UM141 and UM142) after interacting with peritoneal immune cells (24 h). Survival rate was calculated as the ratio between 100 spores plated from both injected and non-injected spores. Statistical analysis was assessed using one-way ANOVA followed by post hoc pairwise comparisons with Welch’s t-test. Significant differences are indicated by asterisks (***p < 0.001, ****p < 0.0001).
(TIFF)

S1 Table Statistical analysis of sporulation and radial growth in *R. microsporus* mutants. The table summarizes mean ± SD values, and the statistical tests performed for each comparison.
(DOCX)

S2 Table Strains of *Rhizopus microsporus* and *Mucor lusitanicus* that were used and generated in this work. (DOCX)

S3 Table Primers used in the mutant generation in *Mucor lusitanicus* and *Rhizopus microsporus*, and for fungal burden quantification. (DOCX)

S4 Table gRNAs used in the mutant generation in *Rhizopus microsporus.* (DOCX)

S1 File Results of differential expression analyses for all comparissons shown in the study. (XLSX)

S2 File Results of differential production of small RNAs (sRNAs) between wild-type and mutant strain confronting macrophages and under saprophytic conditions. (XLSX)

S3 File Results of InterproScan domain prediction for *brac1*, *box*, *hda10* and *hist1* orthologs. (XLSX)
