# Supplementary figures and images for: A BRCT domain-containing protein induced in early phagocytosis plays a crucial role in the pathogenesis of the mucoralean Rhizopus microsporus
Source: PLoS Pathog. 2026 Jan 2;22(1):e1013653. doi: 10.1371/journal.ppat.1013653 (PMC12818731; doi:10.1371/journal.ppat.1013653)

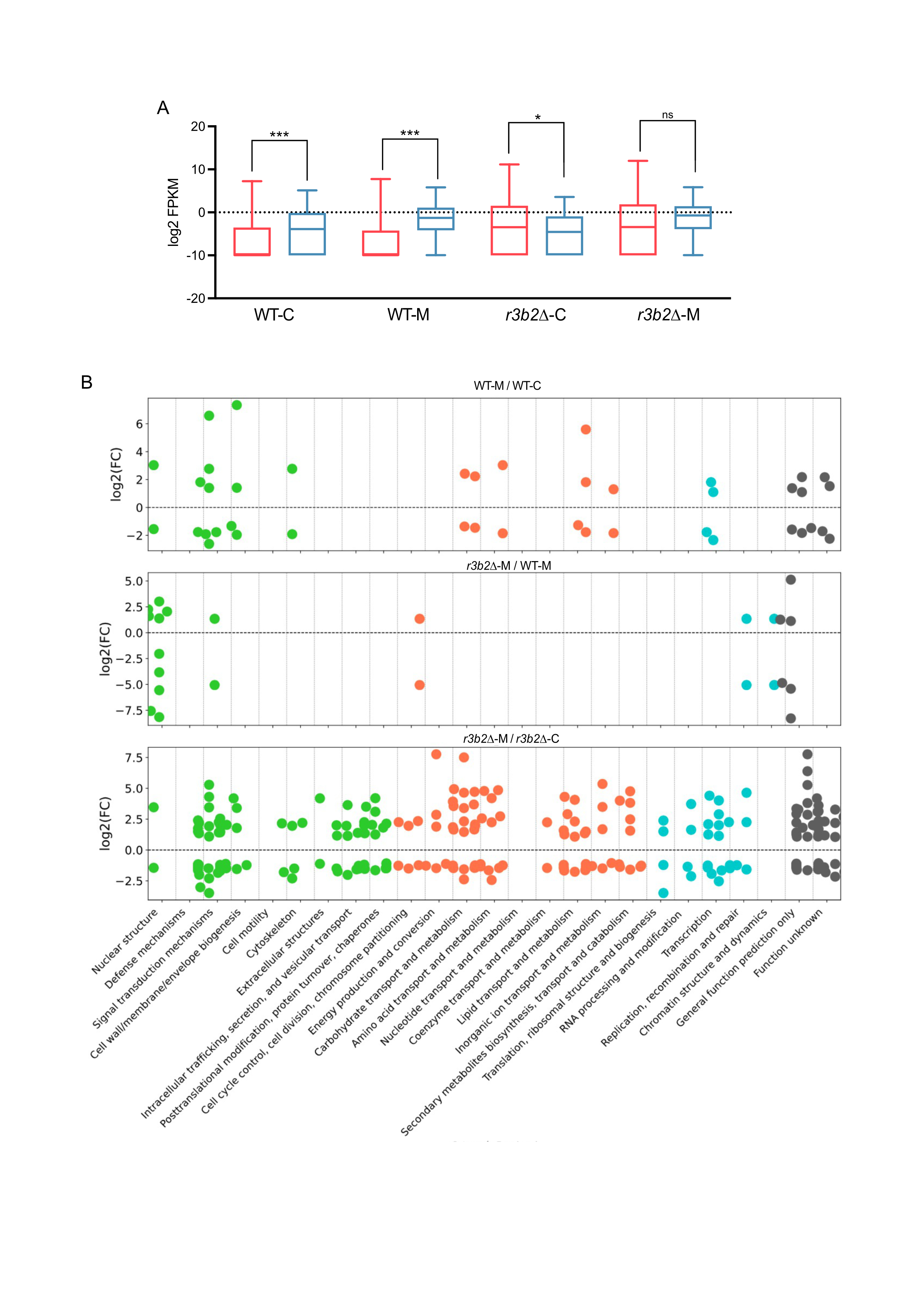

Supplement: S1 Fig — The top 100 genes with the highest and lowest sRNA production were selected for the WT strain with and without macrophages, and the r3b2Δ with and without macrophages. The mRNA expression levels of these top 100 genes, represented as the log2 of FPKM, were plotted for each group. Boxplots indicate the median, first, and third quartiles; whiskers extend to the 10th and 90th percentiles. Statistical significance was assessed using Welch’s t-test. Significance levels: *p < 0.05, ***p < 0.001. (B) Functional analysis of genes showing increased mRNA levels and decreased corresponding sRNAs during interactions of WT and r3b2∆ strains with macrophages across the comparisons: WT-M/ WT-C, r3b2∆-M/ WT-M, and r3b2∆-M/r3b2∆-C). (TIFF) [file ppat.1013653.s001.tiff]

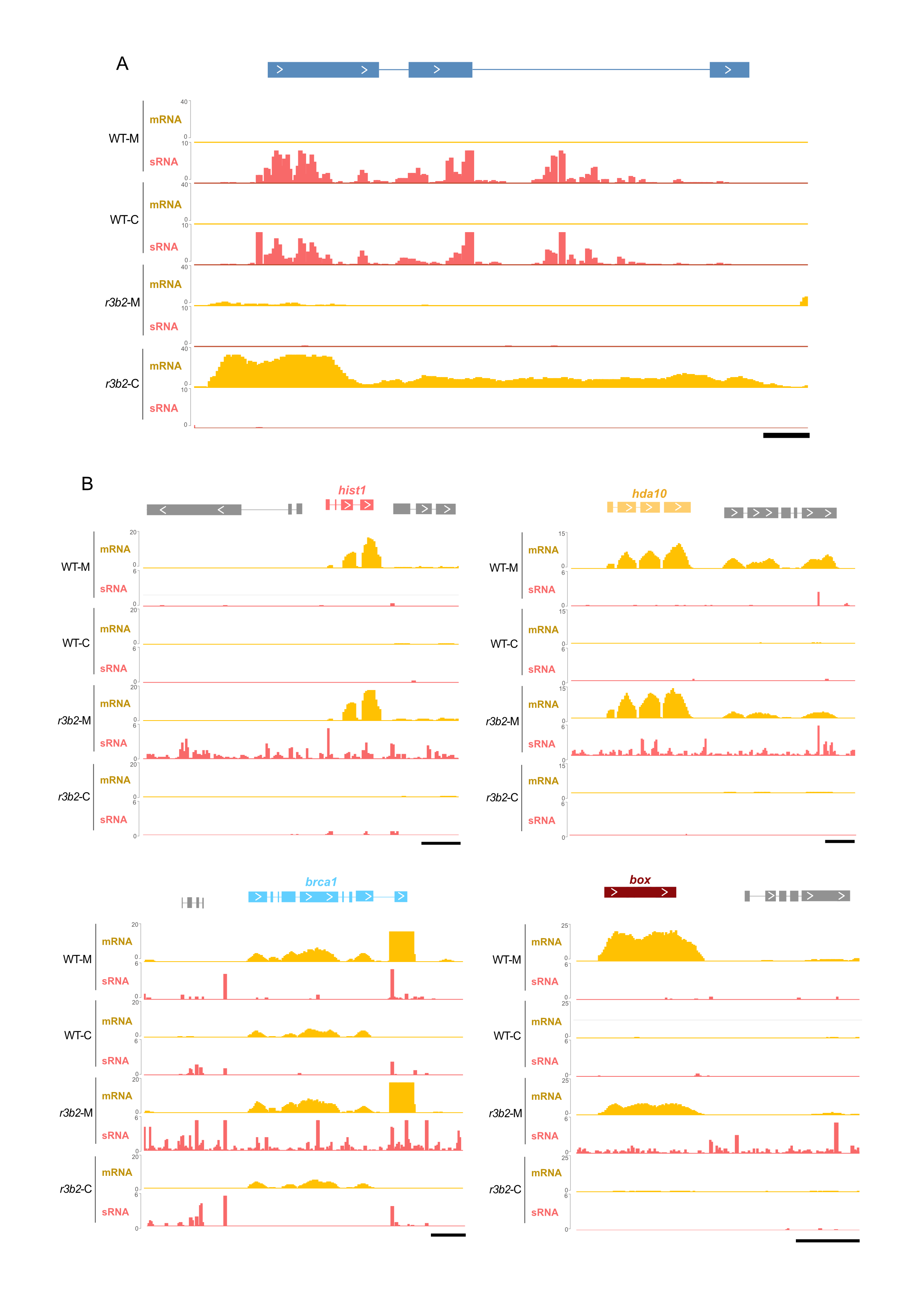

Supplement: S2 Fig — (B) Genomic coverage of sRNA and mRNA reads mapped to the transcription factors brca1 and box, and the chromatin-associated genes hist1 and hda10 in WT and r3b2∆ strains, under saprophytic and macrophage-interacting conditions. Yellow and red plots indicate sRNA and mRNA read coverage, respectively. (TIFF) [file ppat.1013653.s002.tiff]

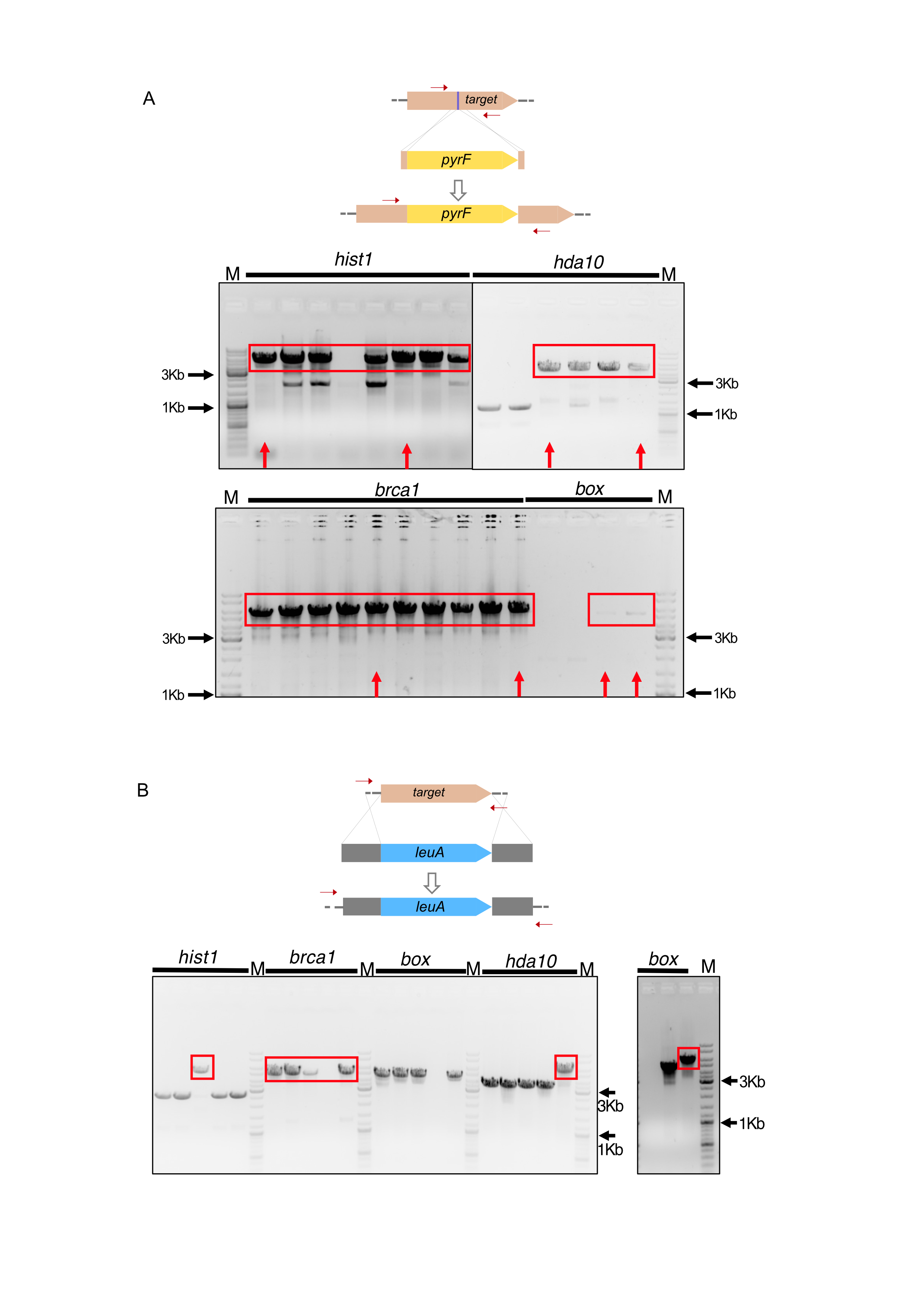

Supplement: S3 Fig — PCRs to check if the mutants are homokaryonts were conducted using a forward and a reverse primers that bind upstream and downstream the regions used for the fusion (Locus_F and Locus_R). The PCR products in the deleted mutant result in 5 Kb, 5 Kb, 5.1 Kb, and 5.3 Kb in the hist1, brca1, hda10, and box loci respectively. The PCR products with the wild-type genes have 0.63 Kb, 4.8 Kb, 3.5 Kb, and 4.3 Kb for hist1, brca1, hda10, respectively. Red boxes show the expected PCR products in the deletion nuclei. The heterokaryons were submitted to further vegetative cycles in selective media. PCRs to check homokaryosis of R. microsporus mutants in phagocytosis-related genes. (B) PCRs to check R. microsporus homokaryosis of the gene candidate disruptions, using specific primers that hybrid approximately 1 Kb from the 38 bp homology regions. The amplification fragments lengths (shown in red boxes) of the disrupted genes are 5.5Kb for hist1, hda10, and brca1 disruptions, and 5.3 Kb for the box disruption. The amplification product lengths resulting from the WT nuclei are 2 Kb for the hist1, hda10, and brca1 genes, and 1.8Kb for the box locus. The mutants containing only nuclei with the disruptions (red rows) were selected for the subsequent analysis. (TIFF) [file ppat.1013653.s003.tiff]

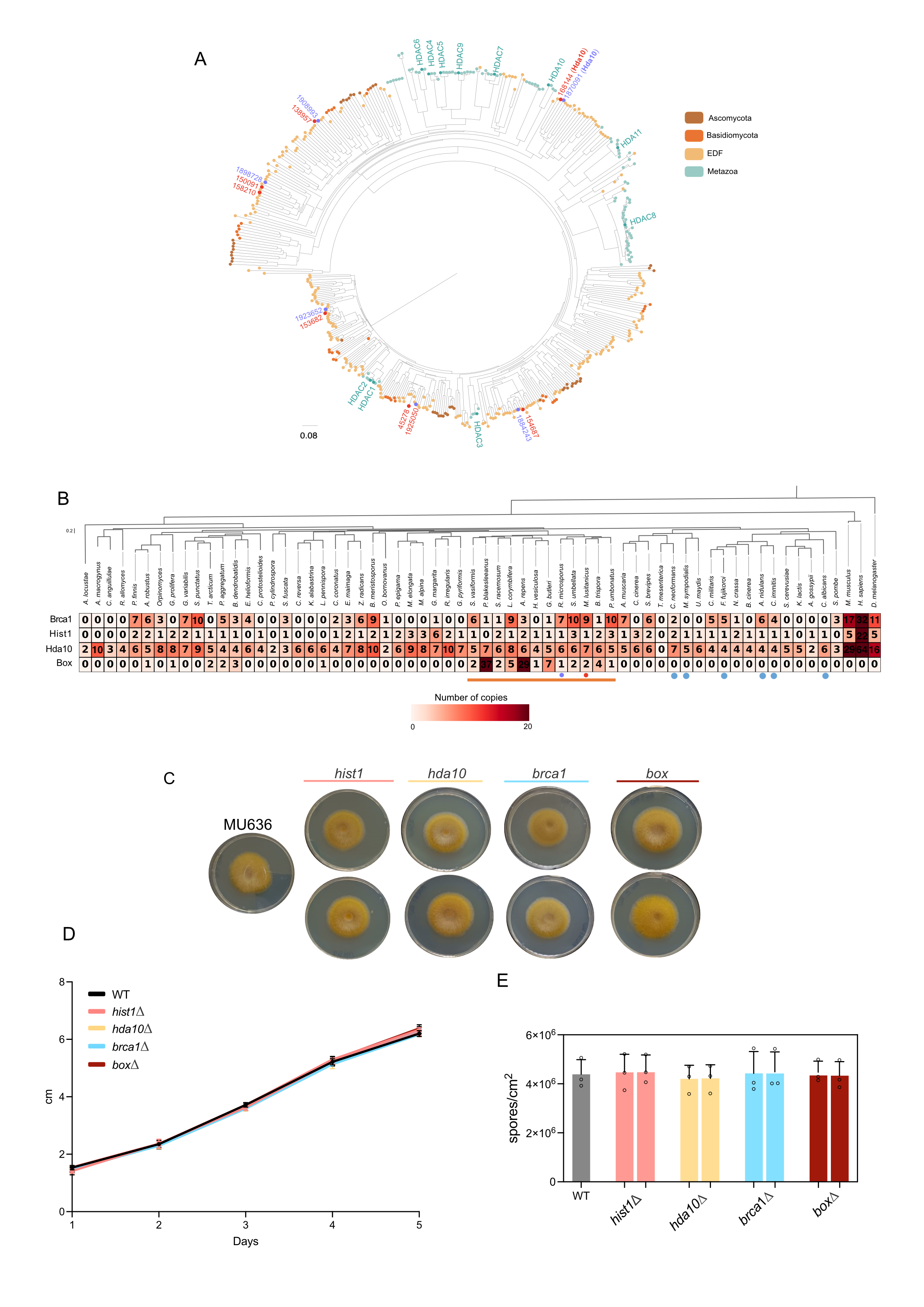

Supplement: S4 Fig — Maximum likelihood phylogeny of histone deacetylase (HDA) proteins from M. lusitanicus (red), R. microsporus (violet), and other representative species from the Early Diverging Fungi (EDF), Ascomycota, Basidiomycota, and Metazoa. Human HDA isoforms were included as outgroups to classify fungal HDAs and infer potential functional conservation. (B) Comparative phylogenomic distribution analysis showing the presence/absence and copy number of Brca1, Box, HDA10, and Histone H1 homologs across multiple fungal genomes. M. lusitanicus, R. microsporus are indicated by red and violet dots, respectively, and other opportunistic human pathogens with blue dots. (C) Sporulation of mutants with deletions in transcriptional regulators and chromatin-related genes in M. lusitanicus. (D) Monitoring of radial growth of the same mutants every 24 hours over 5 days. (E) Spore production per cm² in the WT strain and each mutant after 2 days of growth. Statistical significance was assessed using one-way ANOVA followed by post hoc pairwise comparisons with Welch’s t-test. No asterisks indicate that differences were not statistically significant. (TIFF) [file ppat.1013653.s004.tiff]

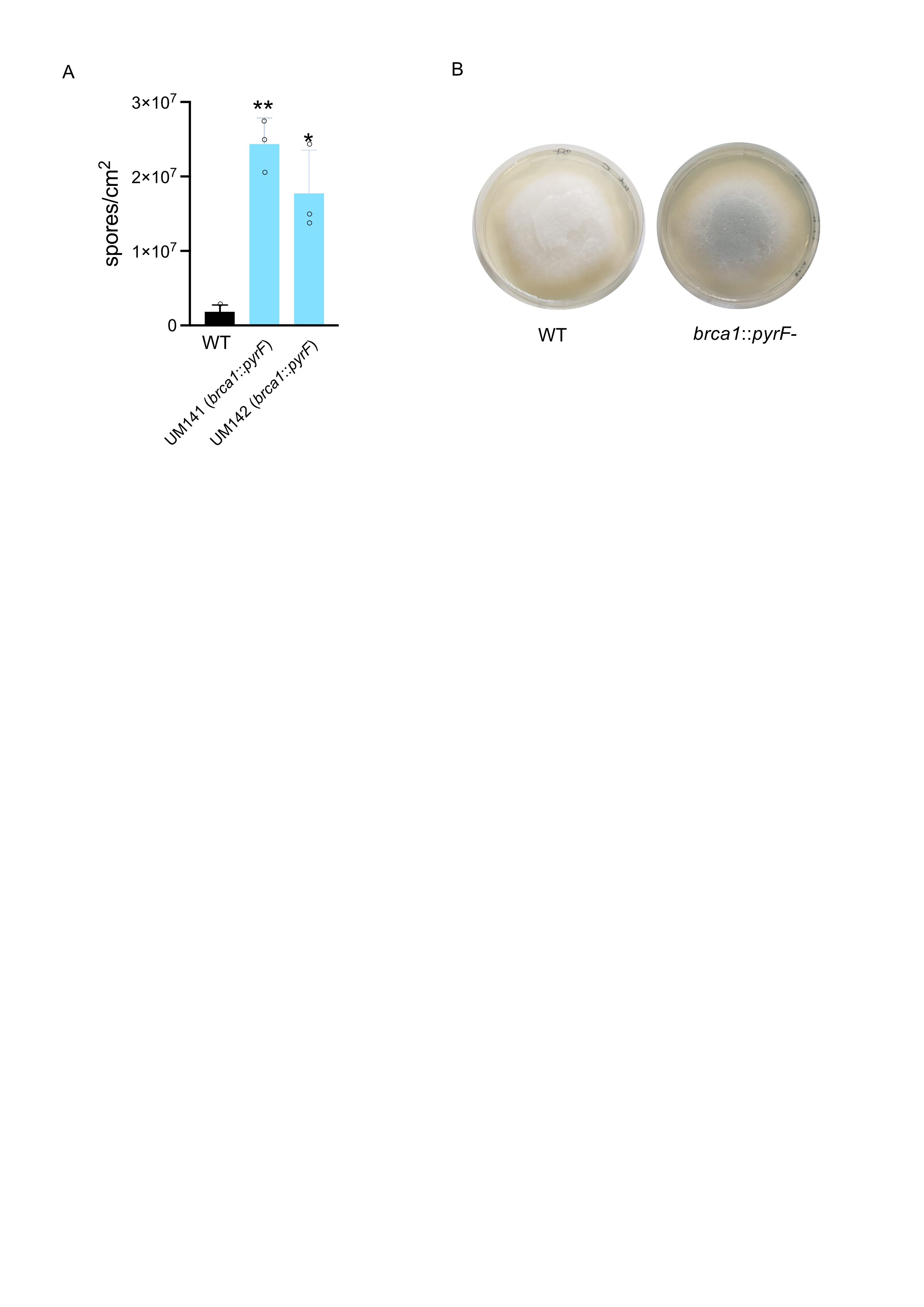

Supplement: S5 Fig — (A) Sporulation of two R. microsporus mutants disrupted in the brca1 gene was evaluated under dark conditions at 37°C. (B) Plates showing sporulation differences between the mutants and WT strain at 37ºC and in darkness. Statistical analysis was assessed using one-way ANOVA followed by post hoc pairwise comparisons with Welch’s t-test. Significant differences are indicated by asterisks (* p < 0.05, ** p < 0.01). (TIFF) [file ppat.1013653.s005.tiff]

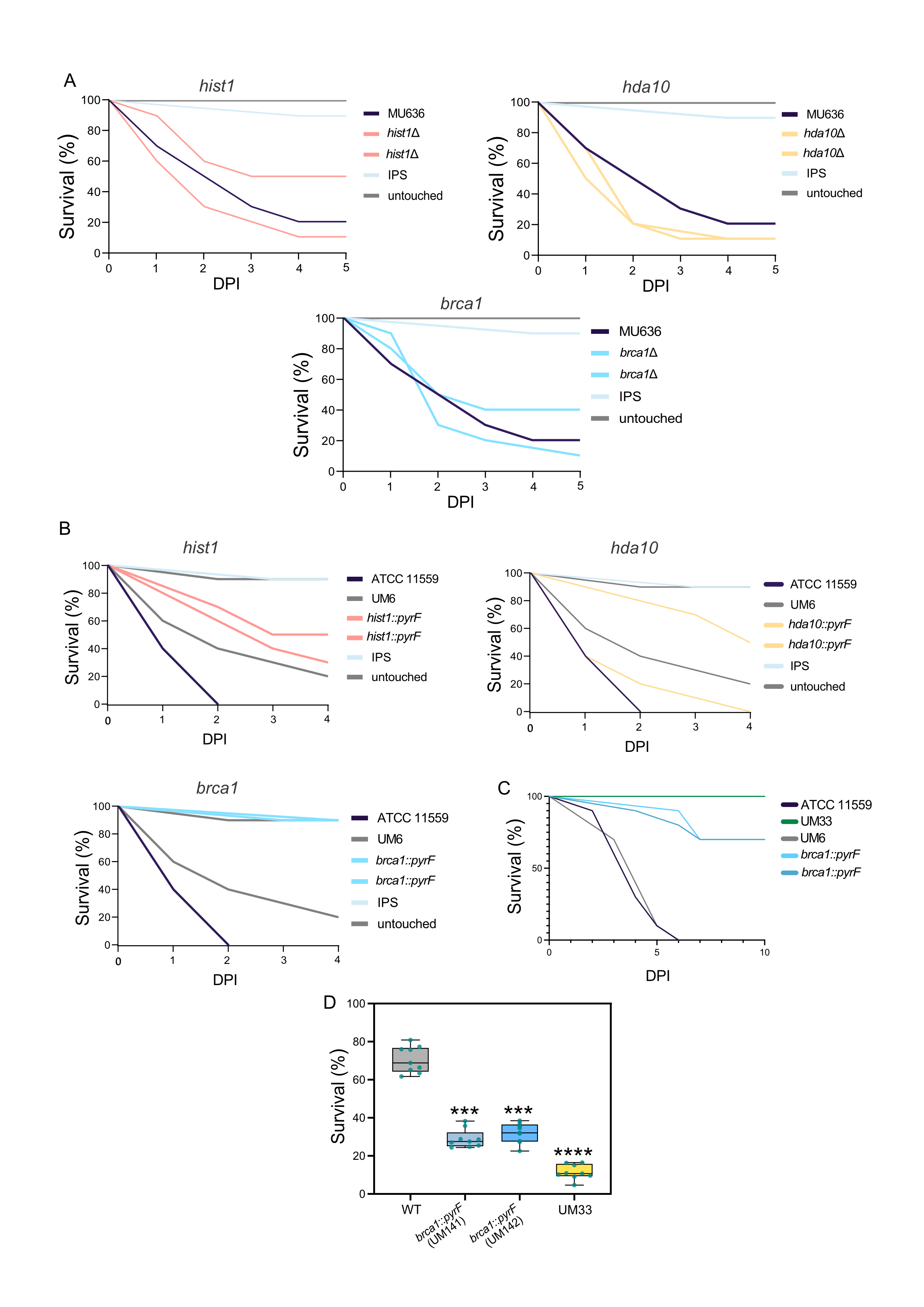

Supplement: S6 Fig — (A) Average survival of G. mellonella larvae infected with M. lusitanicus spores from the indicated mutants and the WT strain. Data represent the mean of three independent experiments. (B) Average survival of G. mellonella larvae infected with R. microsporus spores from the corresponding mutants. (C) Survival of mice infected with R. microsporus spores from two independent brca1 mutants disrupted in brca1, along with virulent and avirulent control strains. Survival was analyzed using the log-rank (Mantel–Cox) test (p ≤ 0.05). DPI, days post-injection. (D) Survival rate of spores from WT, avirulent strain (UM33), and brca1 mutant strains (UM141 and UM142) after interacting with peritoneal immune cells (24 h). Survival rate was calculated as the ratio between 100 spores plated from both injected and non-injected spores. Statistical analysis was assessed using one-way ANOVA followed by post hoc pairwise comparisons with Welch’s t-test. Significant differences are indicated by asterisks (***p < 0.001, ****p < 0.0001). (TIFF) [file ppat.1013653.s006.tiff]
