# Supplementary material for: A BRCT domain-containing protein induced in early phagocytosis plays a crucial role in the pathogenesis of the mucoralean Rhizopus microsporus
Source: PLoS Pathog. 2026 Jan 2;22(1):e1013653. doi: 10.1371/journal.ppat.1013653 (PMC12818731; doi:10.1371/journal.ppat.1013653)
Supplement: S1 Table — The table summarizes mean ± SD values, and the statistical tests performed for each comparison. (DOCX) [file ppat.1013653.s007.docx]

Supplementary Table 1. Statistical analysis of sporulation and radial growth in R. microsporus mutants. The table summarizes mean ± SD values, , and the statistical tests performed for each comparison.

| Experiment | Strain | **mean ± SD** | Welch’s t-test |
| --- | --- | --- | --- |
| Sporulation *R. microsporus* | WT | 4.97 × 10⁷ ± 0.65 × 10⁷ |  |
|  | UM145 | 2.90 × 10⁷ ± 0.25 × 10 | p = 0.027 |
|  | UM146 | 3.23 × 10⁷ ± 0.23 × 10⁷ | p = 0.043 |
| Radial growth *R. microsporus* (Day 1) | WT | 1.70 ± 0.10 cm |  |
|  | UM145 | 1.43 ± 353 0.06 cm | p = 0.025 |
|  | UM146 | 1.27 ± 0.07 cm | p = 0.008 |
| Radial growth *R. microsporus* (Day 5) | WT | 6.67 ± 0.10 cm |  |
|  | UM145 | 6.00 ± 0.06 cm | p = 0.008 |
|  | UM146 | 5.80 ± 0.07 cm | p = 356 0.003 |
| Effect of temperature and light on mutant growth of *R. microsporus* | WT | 1.84 365 × 10⁶ ± 0.12 × 10⁶ |  |
|  | UM141 | 2.44 × 10⁷ ± 0.40 × 10⁷ | p= 0.005 |
|  | UM142 | 1.77 × 10⁷ ± 0.33 × 10⁷ | p= 0.039 |
