# Supplementary material for: A BRCT domain-containing protein induced in early phagocytosis plays a crucial role in the pathogenesis of the mucoralean Rhizopus microsporus
Source: PLoS Pathog. 2026 Jan 2;22(1):e1013653. doi: 10.1371/journal.ppat.1013653 (PMC12818731; doi:10.1371/journal.ppat.1013653)
Supplement: S2 Table — (DOCX) [file ppat.1013653.s008.docx]

Supplementary Table 2. Strains of Rhizopus microsporus and Mucor lusitanicus that were used and generated in this work.

| Strain | Genotype | Description | Organism | Source |
| --- | --- | --- | --- | --- |
| ATCC11559 | WT | WT strain | *R. microsporus* | Lax et al. 2021 |
| UM33 | *pyrF-, leuA-* | Avirulent Control. Used as a receptor strain for the mutant generation | *R. microsporus* | This work |
| UM6 | *pyrF+, leuA-* | Virulent strain | *R. microsporus* | Lax et al. 2021 |
| UM121 | *leuA-, hda10::pyrF* | Mutant in which the *hda10* gene has been disrupted with the *pyrF* gene. Derived from UM33 | *R. microsporus* | This work |
| UM122 | *leuA-, hda10::pyrF* | Mutant in which the *hda10* gene has been disrupted with the *pyrF* gene. Derived from UM33 | *R. microsporus* | This work |
| UM141 | *leuA-, brca1::pyrF* | Mutant in which the *brca1* gene has been disrupted with the *pyrF* gene. Derived from UM33 | *R. microsporus* | This work |
| UM142 | *leuA-, brca1::pyrF* | Mutant in which the brca1 gene has been disrupted with the *pyrF* gene. Derived from UM33 | *R. microsporus* | This work |
| UM143 | *leuA-, box::pyrF* | Mutant in which the *box* gene has been disrupted with the pyrF gene. Derived from UM33 | *R. microsporus* | This work |
| UM144 | *leuA-, box::pyrF* | Mutant in which the *box* gene has been disrupted with the *pyrF* gene. Derived from UM33 | *R. microsporus* | This work |
| UM145 | *leuA-, hist1::pyrF* | Mutant in which the *hist1* gene has been disrupted with the *pyrF* gene. Derived from UM33 | *R. microsporus* | This work |
| UM146 | *leuA-, hist1::pyrF* | Mutant in which the *hist1* gene has been disrupted with the *pyrF* gene. Derived from UM33 | *R. microsporus* | This work |
| R7B | *pyrG+, leuA-* | WT virulent | *Mucor lusitanicus* | Pérez-Arques et al 2019 |
| NRRL3631 | *pyrG+, leuA+* | WT avirulent | *Mucor lusitanicus* | Pérez-Arques et al 2020 |
| MU412 | *pyrG+, leuA-* | Homokaryotic mutant | *Mucor lusitanicus* | Trieu et al. 2015 |
| MU636 | *pyrG+, leuA-* | WT virulent | *Mucor lusitanicus* | Pérez-Arques et al 2019 |
| CBS277.49 | *pyrG+, leuA+* | WT virulent (prototroph) | *Mucor lusitanicus* | Pérez-Arques et al 2020 |
| MU1243 | *pyrG+, leuA+*,  *hda10∆* | Homokaryotic mutant derived from MU636 in which the 168144 (*hda10*) gene has been deleted. Checked by PCR | *Mucor lusitanicus* | This work |
| MU1244 | *pyrG+, leuA+*,  *hda10∆* | Homokaryotic mutant derived from MU636, in which the 168144 (*hda10*) gene has been disrupted. Checked by PCR | *Mucor lusitanicus* | This work |
| MU1246 | *pyrG+, leuA+*,  *hist1∆* | Homokaryotic mutant derived from MU636 in which the 83400 gene (*hist1*) has been disrupted. Checked by PCR | *Mucor lusitanicus* | This work |
| MU1248 | *pyrG+, leuA+*,  *hist1∆* | Homokaryotic mutant derived from MU636 in which the 83400 gene (*hist1*) has been disrupted. Checked by PCR | *Mucor lusitanicus* | This work |
| MU1250 | *pyrG+, leuA+*,  *brca1∆* | Homokaryotic mutant derived from MU636 in which the 113714 (*brca1*) gene has been disrupted. Checked by PCR | *Mucor lusitanicus* | This work |
| MU1252 | *pyrG+, leuA++*,  *brca1∆* | Homokaryotic mutant derived from MU636 in which the 113714 (*brca1*) gene has been disrupted. Checked by PCR | *Mucor lusitanicus* | This work |
| MU1254 | *pyrG+, leuA+*,  *box∆* | Homokaryotic mutant derived from MU636 in which the 104613 (*box*) gene has been disrupted. Checked by PCR | *Mucor lusitanicus* | This work |
| MU1255 | *pyrG+, leuA+*,  *box∆* | Homokaryotic mutant derived from MU636 in which the 104613 (*box*) gene has been disrupted. Checked by PCR | *Mucor lusitanicus* | This work |
