# Supplementary material for: A BRCT domain-containing protein induced in early phagocytosis plays a crucial role in the pathogenesis of the mucoralean Rhizopus microsporus
Source: PLoS Pathog. 2026 Jan 2;22(1):e1013653. doi: 10.1371/journal.ppat.1013653 (PMC12818731; doi:10.1371/journal.ppat.1013653)
Supplement: S3 Table — (DOCX) [file ppat.1013653.s009.docx]

Supplementary Table 3. Primers used in the mutant generation in Mucor lusitanicus and Rhizopus microsporus, and for fungal burden quantification.

|  | Amplified gene | 5’3’ |
| --- | --- | --- |
| q-RT-PCR | 89889  *ltr-transposon*_Fw | GGACGCAATCACTGAACTGC |
|  | 89889  *ltr-transposon*_Rv | TTGGCGGCGTAATTCCTTCAG |
|  | 165343  *permease_*Fw | TTGAGAACCTGGCGGATGAG |
|  | 165343  *permease_*Rv | GACCTTGGCCGATAACACCA |
|  | 83143  *prd_*Fw | TGTCTCGCCTTGCTATCCTG |
|  | 83143  *prd_*Rv | CAGTTGTCGCCGAGTCCATATC |
|  | 113714  *brca1_*Fw | CATCATCGCAACAGCAGCAG |
|  | 113714  *brca1_*Rv | TTCGGTTCTCACGTCCAGAC |
|  | 104613  *boxC_*Fw | TCAGCCAGTACGACACATTC |
|  | 104613  *boxC_*Rv | GGTGATGAAGCGGTTGATGG |
|  | 83400  *hist1*_Fw | CTCGTCCTGCCATCAAGAAG |
|  | 83400  *hist1*_Rv | CACGTCGAATGGCACCAGAG |
|  | 168144  *hda1*_Fw | CGCTTAACAGAGGCCATTGC |
|  | 168144  *hda1*_Rv | GAGCAACAACAGATGCATTG |
| Phagocytosis-related mutants in *Mucor lusitanicus* | BRCA1_1Kb_UP_Fw | CTTGAAGGCAAGATCCTCCTG |
|  | BRCA1_1Kb_UP_Rv | CtGAATAGAGTTGGTAGGGAGCATACGTACTCGGATATAGCTTGGAATAATAAG |
|  | BRCA1_1Kb_Down_Fw | CattttGTACGATTCTGGTCAACTCGACCTTAAATCTCCACATCTAAATGAAC |
|  | BRCA1_1Kb_Down_Rv | GATTGTGACAAAAACCGCATG |
|  | boxC_1Kb_UP_Fw | GCCCAGTAATTGGCATTACATG |
|  | boxCHMG_1Kb_UP_Rv | ctGAATAGAGTTGGTAGGGAGCATACGTACTCGTCATGATCATTTATATCTGATTTAG |
|  | boxC_1kb_Down_Fw | CattttGTACGATTCTGGTCAACTCGACCTTGAAGTCAACTCTTGGGTGATTTTG |
|  | boxC_1kb_Down_Rv | TAAAGCGTGCTCCTGTAGTC |
|  | hist1_1Kb_UP_Fw | TACGAGTGGCTGCTTGAGTAGAG |
|  | hist1_1Kb_UP_Rv | CtGAATAGAGTTGGTAGGGAGCATACGTACTCGTGAGTCGATAATATGAAGAATG |
|  | hist1_1Kb_Down_Fw | cattttGTACGATTCTGGTCAACTCGACCTCCACTGTCAAAGCTTAATGC |
|  | hist1_1Kb_Down_Rv | TTGTTCAGCTTGGGTAGGTC |
|  | hda1_1Kb_UP_Fw | CTTGCATTGTTTGGAATCTTC |
|  | hda1_1Kb_UP_Rv | ctGAATAGAGTTGGTAGGGAGCATACGTACTCGTTTGGTGTTGCCGAGTTGAAG |
|  | hda1_1Kb_Down_Fw | cattttGTACGATTCTGGTCAACTCGACCTTGAACAGCAATGAATAAAAAGC |
|  | hda1_1Kb_Down_Rv | ACAATCCAGGCACAGTGACAAG |
|  | brca1_locusM_Fw | ACTTCTTGGCCTCTTCCATC |
|  | brca1_locusM_Rv | GTAATAAGGCAACCTTAAGCAC |
|  | boxC_locusM_Fw | GTCTGACCGTGTGCACAGTC |
|  | boxC_locusM_Rv | GAATGGTTTCAGCATCGTATTC |
|  | hist1_locusM_Fw | TGCAGCAAAGAGTCCCTTCTAC |
|  | hist1_locusM_Rv | TTGGTATTTGATTTTGTTCAG |
|  | hda1_locusM_Fw | GCATCTATGCGGTTGTTTGAG |
|  | hda1_locusM_Rv | GATCTTCAACAAGATGGGCAAC |
|  | leuAW1_F | CGCCTCATTGAGTCACTGCCAG |
|  | leuAW3_R | GGAACAACCAGCCTCTCTCC |
|  | LeuAFow3kb-Eco10I-XhoI | CCCCCTCGAGTACGTATGCTCCCTACCAACTCTATTC |
|  | LeuARev3Kb-PstI | CCCCCTGCAGGTCGAGTTGACCAGAATCGTAC |
| Phagocytosis-related mutants in *Rhizopus microporus* | pyrF_F_hist1_Fw | GGTGCCCACTTTGATACCCAAGTTGCCGCTGCTATCAATCCTCCATAAGAATTTGACAG |
|  | pyrF_R_hist1_Rv | CTTTAGGAAGTTCAAAGATGCCCTTGGCATGGCCACGCTGATAAAACGAAGATGTGGCTGTC |
|  | pyrF_F_hda1_Fw | TCATTCAAGCCAATGACTATTCCTTAAAACCAATCCTTTCCTCCATAAGAATTTGACAG |
|  | pyrF_R_hda1_Rv | TCTGTTAAAAACTGAATATAGTCTGATGGATGAACCTCTGATAAAACGAAGATGTGGCTGTC |
|  | pyrF_F_boxC_Fw | GAGCTGGTTAAAGGATGGAGTGAATGGTGGCCCTTCATTCCTCCATAAGAATTTGACAG |
|  | pyrF_R_boxC_Rv | TGACTTTTTTGATAACCAGTTGACCAACACATCCATAGTGATAAAACGAAGATGTGGCTGTC |
|  | pyrF_F_brca1_Fw | CCAGAAAACAAAATATTAGAAGGTGTAGTTGCCTGTTTTCCTCCATAAGAATTTGACAG |
|  | pyrF_R_brca1_Rv | AACGTGATCAAGTACATATTGTTACTACCTAACATCTATGATAAAACGAAGATGTGGCTGTC |
|  | hist1_Rz_locus_Fw | TTGATCAGAATGACCTTGGC |
|  | hist1_Rz_locus_Rv | ATCCTATGGTAAGGCCTTCTC |
|  | hda1_Rz_locus_Fw | ATTAAGTATCTGTAATGAAG |
|  | hda1_Rz_locus_Rv | TCCATAAAGCCCTTTAATAC |
|  | brca1__Rz_locus_Fw | GTTACATGAAGGCTATCAAG |
|  | brca1__Rz_locus_Rv | CACAACGGATGCGGATGATG |
|  | boxC_Rz_locus_F | CAGTGATAAAAGGCGCGTAG |
|  | boxC_Rz_locus_R | TTATAGTTTATTCAATTGCTC |
| Fungal burden | B2m-F | TTTTCATCTGTCTTCCCCTGT |
|  | B2m-R | GTATGTATCAGTCTCAGTGGG |
|  | RmCS_F1 | ATGCCAAGTGTGCAACCAAC |
|  | RmCS_R1 | CACAGAGCTGAGGCAAAAAGAC |
