# Supplementary material for: A BRCT domain-containing protein induced in early phagocytosis plays a crucial role in the pathogenesis of the mucoralean Rhizopus microsporus
Source: PLoS Pathog. 2026 Jan 2;22(1):e1013653. doi: 10.1371/journal.ppat.1013653 (PMC12818731; doi:10.1371/journal.ppat.1013653)
Supplement: S4 Table — (DOCX) [file ppat.1013653.s010.docx]

Supplementary Table 4. gRNAs used in the mutant generation in Rhizopus microsporus.

|  | Target gene | 5’3’ |
| --- | --- | --- |
| gRNAs for the phagocytosis related genes in *Rhizopus microsporus* | gRNA_hist1 | GTTGCCGCTGCTATCAAGCG |
|  | gRNA_hda1 | TTCCTTAAAACCAATCCTTG |
|  | gRNA_boxC | GAATGGTGGCCCTTCATCTA |
|  | gRNA_brca1 | TTACTACCTAACATCTAAAC |
